# Divergent selection and drift shape the genomes of two avian sister species spanning a saline–freshwater ecotone

**DOI:** 10.1002/ece3.5804

**Published:** 2019-11-07

**Authors:** Jennifer Walsh, Gemma V. Clucas, Matthew D. MacManes, W. Kelley Thomas, Adrienne I. Kovach

**Affiliations:** ^1^ Department of Natural Resources and the Environment University of New Hampshire Durham NH USA; ^2^ Fuller Evolutionary Biology Program Cornell Laboratory of Ornithology Cornell University Ithaca NY USA; ^3^ Department of Ecology and Evolutionary Biology Cornell University Ithaca NY USA; ^4^ Department of Molecular, Cellular and Biomedical Sciences University of New Hampshire Durham NH USA; ^5^ Hubbard Center for Genome Studies Durham NH USA; ^6^ Present address: Cornell Lab of Ornithology Ithaca NY USA

**Keywords:** adaptation, *Ammospiza caudacutus*, *Ammospzia nelsoni*, demography, ecological divergence, ecological speciation, genomics, tidal marshes

## Abstract

The role of species divergence due to ecologically based divergent selection—or ecological speciation—in generating and maintaining biodiversity is a central question in evolutionary biology. Comparison of the genomes of phylogenetically related taxa spanning a selective habitat gradient enables discovery of divergent signatures of selection and thereby provides valuable insight into the role of divergent ecological selection in speciation. Tidal marsh ecosystems provide tractable opportunities for studying organisms' adaptations to selective pressures that underlie ecological divergence. Sharp environmental gradients across the saline–freshwater ecotone within tidal marshes present extreme adaptive challenges to terrestrial vertebrates. Here, we sequence 20 whole genomes of two avian sister species endemic to tidal marshes—the saltmarsh sparrow (*Ammospiza caudacutus*) and Nelson's sparrow (*A. nelsoni*)—to evaluate the influence of selective and demographic processes in shaping genome‐wide patterns of divergence. Genome‐wide divergence between these two recently diverged sister species was notably high (genome‐wide *F*_ST_ = 0.32). Against a background of high genome‐wide divergence, regions of elevated divergence were widespread throughout the genome, as opposed to focused within islands of differentiation. These patterns may be the result of genetic drift resulting from past tidal march colonization events in conjunction with divergent selection to different environments. We identified several candidate genes that exhibited elevated divergence between saltmarsh and Nelson's sparrows, including genes linked to osmotic regulation, circadian rhythm, and plumage melanism—all putative candidates linked to adaptation to tidal marsh environments. These findings provide new insights into the roles of divergent selection and genetic drift in generating and maintaining biodiversity.

## Graphical abstract

Whole‐genome comparisons of two congeneric sparrows with different levels of specialization to tidal marsh environments revealed that both genetic drift and divergent ecological selection influenced the speciation process. Against a background of high genome‐wide divergence brought about by drift, regions of elevated divergence were widespread throughout the genome. These regions contained genes relating to tidal marsh adaptations, including those putatively functioning in osmotic regulation, circadian rhythm, and plumage melanism, and suggests a role for ecological speciation in the divergence of the saltmarsh and Nelson's sparrow.

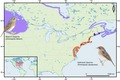

## 1 INTRODUCTION

The role of differential adaptation to divergent selective environments in generating and maintaining biodiversity has become an increasing focus for evolutionary biologists over the past decade and has been termed ecological speciation (Funk, Nosil, & Etges, 2006; Rundle & Nosil, 2005; Schluter, 2001). While the concept of ecologically based divergent selection is not new (Mayr, 1942; Rundle & Nosil, 2005), disentangling the contribution of ecological forces from nonecologically based evolutionary forces (i.e., reductions in population size and genetic drift) remains a challenge. Despite these challenges, increasing accessibility and improvement of current sequencing technologies have allowed for the application of whole‐genome sequencing to questions in natural populations (Campagna et al., 2017; Ellegren, 2014; Toews et al., 2016). The genomic era holds promise for ecological speciation research, as it has allowed for the detection of divergent signatures of selection on a genome‐wide scale. In recent years, the application of genomics to nonmodel systems has provided new insight into mechanisms underlying lineage‐specific adaptations in a range of taxa (Cai et al., 2013; Li, Li, Jia, Caicedo, & Olsen, 2017; Liu et al., 2015; Qiu et al., 2012; Zhan et al., 2013). It has also provided insight into the genomic architecture of adaptation and speciation (Cruickshank & Hahn, 2014; Larson et al., 2017; Strasburg et al., 2012).

Under a scenario of ecological speciation, divergent selection will result in differential performance of individuals inhabiting alternative ecological niches (Arnegard et al., 2014; Nosil, 2012). Reproductive isolation resulting from such adaptive divergence may occur either in sympatry, parapatry, or allopatry (Langerhans, Gifford, & Joseph, 2007; Nosil, 2007, 2012). Comparison of the genomes of two ecologically divergent taxa spanning a selective habitat gradient provides valuable insight into the role of niche divergence in driving natural selection and speciation within a system. Characterizing genomic differentiation in recently diverged taxa is critical for increasing understanding of ecological speciation, as early acting barriers to gene flow have a larger effect on driving reproductive isolation than late‐acting barriers (Coyne & Orr, 2004). In addition, identifying elevated regions of differentiation—and potential genomic regions under selection—is easier when baseline divergence is low, as expected for phylogenetically closely related and recently diverged taxa (Campagna et al., 2017; Ellegren et al., 2012; Poelstra et al., 2014; Toews et al., 2016).

Tidal marsh habitats in North America have undergone rapid changes since the last glacial maximum (Greenberg, 2006), and they are comprised of sharp ecotones that provide highly tractable opportunities for understanding underlying spatial patterns of genetic variation and adaptation. Because most tidal marsh endemics have colonized these habitats only after the rapid expansion of coastal marshes approximately 5,000–7,000 years ago (Malamud‐Roam, Malamud‐Roam, Watson, Collins, & Ingram, 2006), these systems provide opportunities to investigate patterns of recent and contemporary evolution. Environmental gradients across the saline–freshwater ecotone present extreme adaptive challenges to terrestrial vertebrates (Bayard & Elphick, 2011; Greenberg, 2006). Divergent selection among populations spanning these salinity gradients can be apparent in both physiological (i.e., pathways involved in osmotic regulation) and morphological (i.e., plumage—saltmarsh melanism, bill, and body size variation; Grenier & Greenberg, 2005; Grinnell, 1913) traits. These strong ecological gradients in tidal marshes provide a model system for applying comparative genomic analysis to investigate the role of ecological divergence in shaping species diversity.

Here, we investigated patterns of genome‐wide differentiation between two recently diverged marsh endemics, the saltmarsh (*Ammospiza caudacutus*) and Nelson's (*A. nelsoni*) sparrow (~600,000 years; Rising & Avise, 1993). Although long considered a single species (AOU, 1931), Nelson's and saltmarsh sparrow are currently recognized as two species comprised of a total of five subspecies: *A. nelsoni nelsoni*—breeds in the continental interior from eastern British Columbia to central Manitoba and northern South Dakota; *A. nelsoni alterus*—around the James and Hudson bays; *A. nelsoni subvirgatus*—across the Canadian Maritimes to southern Maine; *A. caudacutus caudacutus*—from southern Maine to New Jersey; and *A. caudacutus diversus*—from southern New Jersey to Virginia (Greenlaw & Rising, 1994). The prevailing evolutionary hypothesis (Greenlaw, 1993) suggests a history of vicariance for the saltmarsh and Nelson's sparrow, whereby an ancestral population spanning a coastal to interior range was split by Pleistocene glaciation, resulting in an isolated interior population. Following differentiation, this interior population then spread eastward back toward the Atlantic coast after recession of the Wisconsin ice mass, making secondary contact with ancestral coastal populations and establishing the current ranges and ecotypes within this species complex (Greenlaw, 1993). Recent analyses of genetic and morphological characters indicate the strongest differences appear to correspond to habitat type, clustering the five subspecies into three groups: (a) the two freshwater, interior subspecies of Nelson's sparrow; (b) the brackish, coastal subspecies of Nelson's sparrow; and (c) the two saltwater, coastal subspecies of saltmarsh sparrow (Walsh et al., 2017).

While both species inhabit tidal marshes in sympatry, variation in habitat affinity, morphology, and behavior suggest a role for divergent selection and adaptation in this system. Specifically, the saltmarsh sparrow is a narrow niche specialist that has been associated with salt marshes over a longer evolutionary time frame (possibly 600,000 years, Chan, Hill, Maldonado, & Fleischer, 2006) compared with Nelson's sparrow, which uses a broader range of habitats including brackish and freshwater marshes and hayfields (Greenlaw, 1993; Nocera, Fitzgerald, Hanson, & Milton, 2007; Shriver, Hodgman, & Hanson, 2011). Further, Nelson's sparrow has lower nesting success in tidal, coastal marshes compared with saltmarsh sparrow, suggesting habitat‐linked adaptive differences (Maxwell, 2018; Shriver, Vickery, Hodgman, & Gibbs, 2007; Walsh, Rowe, Olsen, Shriver, & Kovach, 2015). Due to these differences in niche specificity, differentiation of these sister species may have been largely driven by divergent natural selection. Alternatively, changes in population size during vicariant isolation and colonization events may have increased the role of genetic drift in driving interspecific divergence. While previous work in this system has identified patterns consistent with divergent selection across the saline–freshwater ecotone (Walsh et al., 2017, 2015; Walsh, Shriver, Olsen, & Kovach, 2016), the genome‐wide pattern of differentiation between saltmarsh and Nelson's sparrows remains unknown. An understanding of the genomic landscape of these taxa will reveal the influence of demographic processes and divergent selection in ecological speciation.

We sequenced whole genomes of saltmarsh and Nelson's sparrows to investigate the role of divergent selection across an ecological gradient in shaping genome‐wide patterns of divergence. We were interested in identifying genomic regions exhibiting elevated divergence due to selection across the saline–freshwater ecotone. We predicted elevated divergence between saltmarsh and Nelson's sparrows in gene regions linked to known tidal marsh adaptations. Specifically, we hypothesized that genes linked to kidney development, osmotic regulation (salt tolerance in tidal environments; Goldstein, 2006; Greenberg, 2006), circadian rhythm (important for nest initiation relative to tidal cycles; Shriver et al., 2007; Walsh et al., 2017), bill size (larger bills facilitate evaporative heat loss; Greenberg, Danner, Olsen, & Luther, 2012; Greenberg & Danner, 2012; Luttrell, Gonzalez, Lohr, & Greenberg, 2014), and melanic plumage (example of salt marsh melanism; Greenberg & Droege, 1990; Grinnell, 1913; Walsh et al., 2016) would be targets of selection in tidal marsh environments and would be key mechanisms underlying ecological divergence between these species.

## 2 METHODS

### 2.1 Sample collection

**Figure 1:**
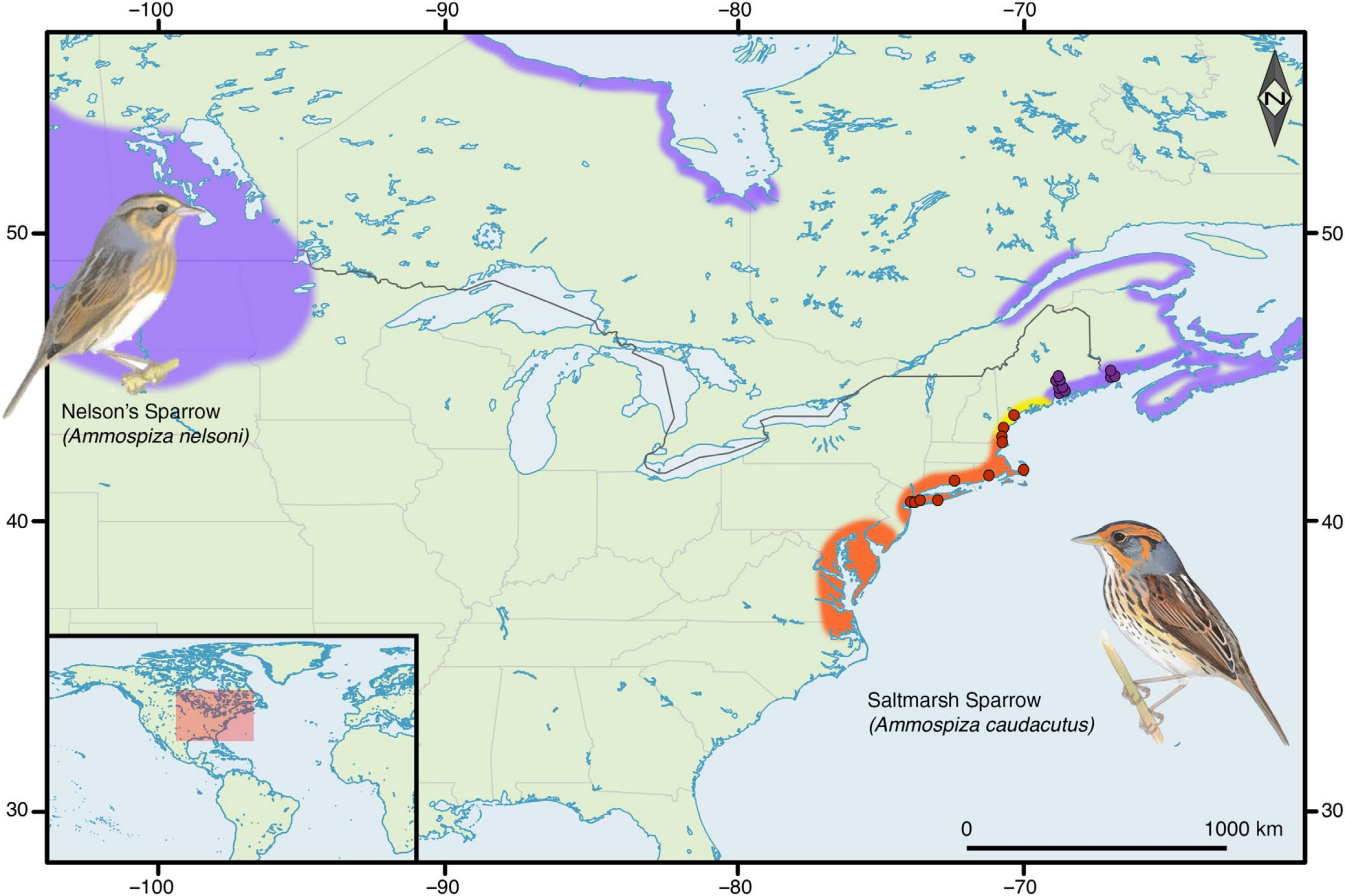
Breeding distributions and sampling locations. The breeding distributions of Nelson's and saltmarsh sparrows are shown in purple and orange, respectively. The hybrid zone along the New England coastline is shown in yellow. The breeding locations of Nelson's sparrows that were resequenced in this study are shown by the purple dots, the resequenced saltmarsh sparrows are shown by the orange dots, and the breeding location of the reference genome individual is shown in dark brown. The inset figures show the plumage of the two species

For the reference genome, we sampled a male saltmarsh sparrow from the Marine Nature Center in Oceanside, New York, in July 2016. Blood was collected from the brachial vein and stored in Puregene lysis buffer (Gentra Systems, Minneapolis, MN). For genome resequencing, we sampled 20 individuals, 10 Nelson's sparrows (2 females and 8 males) and 10 saltmarsh sparrows (8 females and 2 individuals of unknown sex), from marshes along the northeastern coastline of the United States (Figure 1; Table S1) during the breeding seasons (June–August) of 2008–2014. Nelson's sparrows were sampled from three populations in Maine. Saltmarsh sparrows were sampled from ten populations in Massachusetts, Rhode Island, Connecticut, New York, Maine, and New Hampshire. From each bird, we collected blood samples from the brachial vein and stored them on Whatman filter cards.

### 2.2 Reference genome—library construction and assembly

For reference genome sequencing, DNA was extracted from blood stored in lysis buffer using a PureGene extraction kit (Gentra). Three libraries were constructed using an Illumina Nextera library preparation kit: one paired‐end (PE) library with a 180 bp insert size and two mate‐pair (MP) libraries with 3 and 8 kbp insert sizes. Each library was sequenced on a single HiSeq 2500 lane PE x 100 cycles by the Weill Cornell Medical College Core Genomics facility.

Genome assembly was performed with ALLPATHS‐LG version 44849 (Gnerre et al., 2011; Ribeiro et al., 2012) using the default parameters. ALLPATHS‐LG takes raw data as input, without prior adapter removal and trimming. We used bioanalyzer results to estimate the insert size and expected standard deviation, which are required input for ALLPATHS‐LG. The assembly was completed in six days on a 64‐core computer (1,024 GB RAM, 19 TB hard drive) from the Cornell Computational Biology Service Unit BioHPC Lab. We obtained assembly statistics with Quast version 2.3.

### 2.3 Reference genome—annotation

We annotated the saltmarsh sparrow assembly with the MAKER pipeline v 2.31.9 (Cantarel et al., 2008). Gene models were created using the zebra finch (*Taeniopygia guttata*) Ensemble protein database (downloaded 2 March 2017 from http://useast.ensembl.org/Taeniopygia_guttata/Info/Index?redirect=no) and a saltmarsh sparrow transcriptome. To generate the transcriptome, cDNA libraries were prepared from RNA extracted with a RNeasy kit (Qiagen, Valencia, CA, USA) from six tissues after overnight freezing—heart, muscle, liver, brain, gonad, and kidney—from a single male saltmarsh sparrow individual. Sequencing libraries were generated from polyA‐enriched mRNA using the Illumina TruSeq RNA sample prep LT system. RNA sequencing of 100 bp PE reads was performed in a single Illumina HiSeq 2000 lane at Vanderbilt University. Transcriptome assembly was performed on the combined datasets with CLC Genomics Workbench (V5.1.2.) using CLC Assembly Cell 4.0 set to default parameters. Genes were predicted with SNAP v2013‐11‐29, using an iterative training process inside MAKER v2.31.9, and Augustus v3.2.2_4, using a hidden Markov model from the chicken. This produced a total of 15,414 gene models, which included 85.2% complete BUSCO v2.0 (Simão, Waterhouse, Ioannidis, Kriventseva, & Zdobnov, 2015) genes and a further 9.3% which were fragmented, when assessed against the aves_odb9 lineage data and specifying the white‐throated sparrow (*Zonotrichia albicollis*) as the most closely related species in the database. The saltmarsh sparrow assembly was aligned to the zebra finch genome using *Satsuma* Chromosembler (Grabherr et al., 2011). Using this approach, scaffolds were mapped to chromosome coordinates via synteny. Thus, scaffolds were assigned to chromosomes for removal of sex‐linked markers; however, population genetic analyses were conducted at the scaffold level.

### 2.4 Whole‐genome resequencing and variant discovery

We sequenced the genomes of an additional 20 individuals, 10 saltmarsh sparrows and 10 Nelson's sparrows. DNA was extracted using the DNeasy Blood and Tissue Kit (Qiagen). For 7 of the 20 individuals (six saltmarsh and one Nelson's sparrow), we prepared individually barcoded Illumina TruSeq DNA libraries, from 1.2 to 31 µg of DNA, which were sequenced on a Illumina HiSeq 2000 at Vanderbilt University. DNAs from one saltmarsh sparrow (31 µg) and one Nelson's sparrow (6.2 µg) individuals were sequenced each in a single lane, and five saltmarsh sparrows (1.2–3 µg DNA) were sequenced together in a third lane. For the remaining 13 individuals, we prepared individually barcoded libraries using 1–2 ng of DNA following the Nextera^®^ DNA library preparation kit protocol, with a target insert size of 550 bp. We pooled the 13 libraries using concentrations of adapter‐ligated DNA determined through digital PCR and sequenced them as 150‐bp PE reads in two lanes on an Illumina HiSeq 2500 at the University of New Hampshire Hubbard Center for Genome Studies. The quality of individual libraries was assessed using FastQC version 0.11.5 (http://www.bioinformatics.babraham.ac.uk/projects/fastqc).

We used a combination of programs to perform sequence trimming, adapter removal, and quality filtering. These included seqtk v 1.1‐r91 (https://github.com/lh3/seqtk/blob/master/README.md) and Skewer v 0.1.127 (Jiang, Lei, Ding, & Zhu, 2014). We allowed a minimum Phred quality score of 20 and merged overlapping paired‐end reads in Skewer. Filtered reads were aligned to the saltmarsh sparrow reference genome using *bwa‐*mem 0.7.4 (Li & Durbin, 2009) with default settings. Alignment statistics were obtained using qualimap version 2.1.1 (Okonechnikov, Conesa, & García‐Alcalde, 2015). The average alignment rate was 97.3 ± 0.90 and 97.8 ± 0.43 across all saltmarsh and Nelson's sparrow individuals, respectively. After aligning sequences to the saltmarsh sparrow reference genome, depth of coverage ranged from 5 to 34X (18.7 and 34X for the two individuals sequenced in single lanes and 5–8X for the remainder that were multiplexed for sequencing; Table S1).

BAM files were sorted and indexed using Samtools version 1.3 (Li et al., 2009), and PCR duplicates were marked with Picard Tools version 2.1.1 (http://picard.sourceforge.net). We realigned around indels and fixed mate pairs using GATK version 3.5 (McKenna et al., 2010). SNP variant discovery and genotyping for the 20 resequenced individuals were performed using the unified genotyper module in GATK. We used the following filtering parameters to remove variants: QD < 2, FS > 40.0, MQ < 20.0, and HaplotypeScore > 12.0. In addition, variants that were not biallelic, had minor allele frequencies <5%, mean coverage less than 3X or with coverage greater than two standard deviations above the mean, and more than 20% missing data across all individuals were filtered from the data set. This resulted in 7,240,443 SNPs across the genome; the mean SNP depth averaged over individuals was 10.7 (standard deviation: 11.2). Using mapping results from Satsuma, we removed all SNPs located on the Z chromosome to avoid any bias that may be introduced by analyzing a mix of male and female individuals, resulting in a final dataset containing 6,256,980 SNPs.

### 2.5 Population genomics

Principle component analysis (PCA) was performed on all SNPs using the snprelate package in R (R Development Core Team, 2017). We identified divergent regions of the genome by calculating *F*_ST_ values for nonoverlapping 100 kb using VCFtools version 0.1.14 (Danecek et al., 2011). Descriptive statistics for the 100 kb windows, including the number of fixed SNPs, the proportion of fixed SNPs in coding regions, nucleotide diversity (π), Tajima's *D*, mean observed heterozygosity (*H*_obs_), and mean expected heterozygosity (*H*_exp_), were calculated using VCFtools and R. We calculated absolute divergence (*D*_xy_) using a custom python script (S. Martin, https://github.com/simonhmartin/genomics_general). Divergent peaks were visualized using Manhattan plots, which were constructed using the R package qqman. We discarded regions with less than two windows and windows with less than 10 SNPs.

### 2.6 Characterizing divergence between Nelson's and saltmarsh sparrows

To fully assess genome‐wide patterns of differentiation between saltmarsh and Nelson's sparrows and to identify potential genes that play a role in adaptive divergence, we employed a multistep approach to identifying and characterizing candidate regions of interest. First, we defined candidate genomic regions under selection if they contained window‐based *F*_ST_ estimates above the 99th percentile of the empirical distribution. Second, we estimated the density of fixed SNPs (*df*) in the same 100‐kb windows following Ellegren et al. (2012) and identified windows above the 99th percentile of the *df* distribution. For both approaches, elevated windows were inspected in Genious version 9.1.5 (Kearse et al., 2012). We compiled a list of gene models within 50 kb of each elevated region and obtained information on these annotations from the UniProt database (http://www.uniprot.org/). We performed GO analyses of divergent windows (Table 1) using the Web‐based GOfinch tool (http://bioinformatics.iah.ac.uk/tools/Gofinch). Lastly, we compiled a list of candidate genes hypothesized to be important for tidal marsh adaptations, including genes linked to reproductive timing (circadian rhythm genes), osmotic regulation, salt marsh melanism, and bill morphology. Genes were chosen based on previous research done in this system (Walsh et al., 2017, 2016) and a literature review. We estimated *F*_ST_ and *df* for each candidate gene of interest plus 20 kb upstream and downstream of the gene, and compared our divergence estimates to the genome‐wide average.

**Table 1:** Candidate genes linked to tidal marsh adaptations

Scaffold	Window start position	Number of variants	Mean *F*_st_	Number of fixed SNPs	Candidate gene	Putative adaptive function
scaffold_10	1,300,001	657	0.602402	223	MSMB	Specific receptors for this protein are found on spermatozoa
scaffold_10	1,300,001	657	0.602402	223	NPY4R	Blood circulation, chemical synaptic transmission, digestion, feeding behavior, neuropeptide signaling pathway
scaffold_10	1,300,001	657	0.602402	223	WASHC2C	Negative regulation of barbed‐end actin filament capping, protein transport, regulation of substrate adhesion‐dependent cell spreading, retrograde transport
scaffold_11	600,001	316	0.585958	144	ATP1B1	Catalyzes the hydrolysis of ATP coupled with the exchange of Na+ and K+ ions across the plasma membrane
scaffold_11	700,001	288	0.66892	144	SLC19A2	High‐affinity transporter for the intake of thiamine
scaffold_115	500,001	226	0.742673	147	CAMK2D	Cellular response to calcium ion, MAPK cascade, negative regulation of sodium ion transmembrane transport, nervous system development, regulation of cellular response to heat, calmodulin‐dependent protein kinase activity, sodium channel inhibitor activity
scaffold_115	500,001	226	0.742673	147	NPFFR2	Cellular response to hormone stimulus, regulation of cAMP‐dependent protein kinase activity, regulation of MAPK cascade
scaffold_115	500,001	226	0.742673	147	OVGP1	Negative regulation of binding sperm to zona pellucida, single fertilization
scaffold_115	700,001	235	0.628877	127	ALB	Sodium‐independent organic ion transport, retina homeostasis, bile acid, and bile salt transport
scaffold_115	700,001	235	0.628877	127	ANKRD17	Blood vessel maturation, defense response to bacterium, innate immune response, negative regulation of smooth muscle cell differentiation, regulation of DNA replication
scaffold_115	900,001	243	0.673404	130	CXCL5	Immune response, inflammatory response
scaffold_115	900,001	243	0.673404	130	CCNI	Spermatogenesis and regulation of cell cycle
scaffold_115	1,800,001	418	0.594823	144	PCDH18	Brain development, cell adhesion, homophilic cell adhesion, nervous system development
scaffold_215	100,001	238	0.693515	142	ANO2	Calcium‐activated chloride channel (CaCC) which may play a role in olfactory signal transduction
scaffold_261	1	368	0.534709	141	KRTCAP3	Keratinocyte‐associated protein 3, integral component of membrane
scaffold_35	4,500,001	830	0.530141	203	KCNS1	Potassium ion transport
scaffold_35	4,500,001	830	0.530141	203	DTNBP1	Regulates dopamine receptor signaling pathway, blood coagulation, melanosome organization, and neuronal development
scaffold_44	5,700,001	209	0.721474	131	CRY1	Transcriptional repressor which forms a core component of the circadian clock
scaffold_44	5,700,001	209	0.721474	131	TMEM263	Transmembrane protein
scaffold_44	6,400,001	229	0.766646	156	SLC41A2	Acts as a plasma‐membrane magnesium transporter

Note

Candidate regions are housed under windows exhibiting elevated divergence, assessed as regions with both *F*_ST_ estimates and *df* estimates higher than the 99th percentile of the empirical distribution. Table includes information on location (scaffold and window start position), the number of SNPs contained within each window, mean *F*_ST,_ the number of fixed SNPs, gene name, and putative adaptive function. The full list of genes housed in elevated gene regions is found in Table S7.

### 2.7 Modeling demographic history

An investigation of population size history was performed with the pairwise sequentially Markovian coalescent (PSMC) model (Li & Durbin, 2011). We used a single individual from each species, each with mean coverage ≥18X and missing data < 1%. Coverage ≥ 18X and <20% missing data have been shown to be important for accurate inference using PSMC (Nadachowska‐Brzyska, Burri, Smeds, & Ellegren, 2016). Samtools and bcftools were used to create a diploid consensus genome for each individual. For the PSMC inference, we used time intervals denoted by −*p* “60*1 + 2 + 2” meaning that the first sixty atomic time intervals are of length 1, followed by two intervals with length 2. All other parameters were kept as defaults. We ensured that at least 10 coalescent events occurred in each interval after 20 iterations and performed 100 bootstrap replicates. For plotting, we used a mutation rate of 7.59 × 10^−9^ subs/site/generation and a generation time of 2.3 years. The mutation rate was calculated using the mean mutation rate of passeriformes, which was estimated to be 3.3 × 10^−3^ substitutions per site per million years (Zhang et al., 2014). Generation time was estimated using the equation 1/m + b where m is mortality, estimated to be approximately 0.5 for adults and one and a half times that for juveniles, and b, the age of first breeding, is equal to 1 (Greenlaw, Elphick, Post, & Rising, 2018).

**Figure 2:**
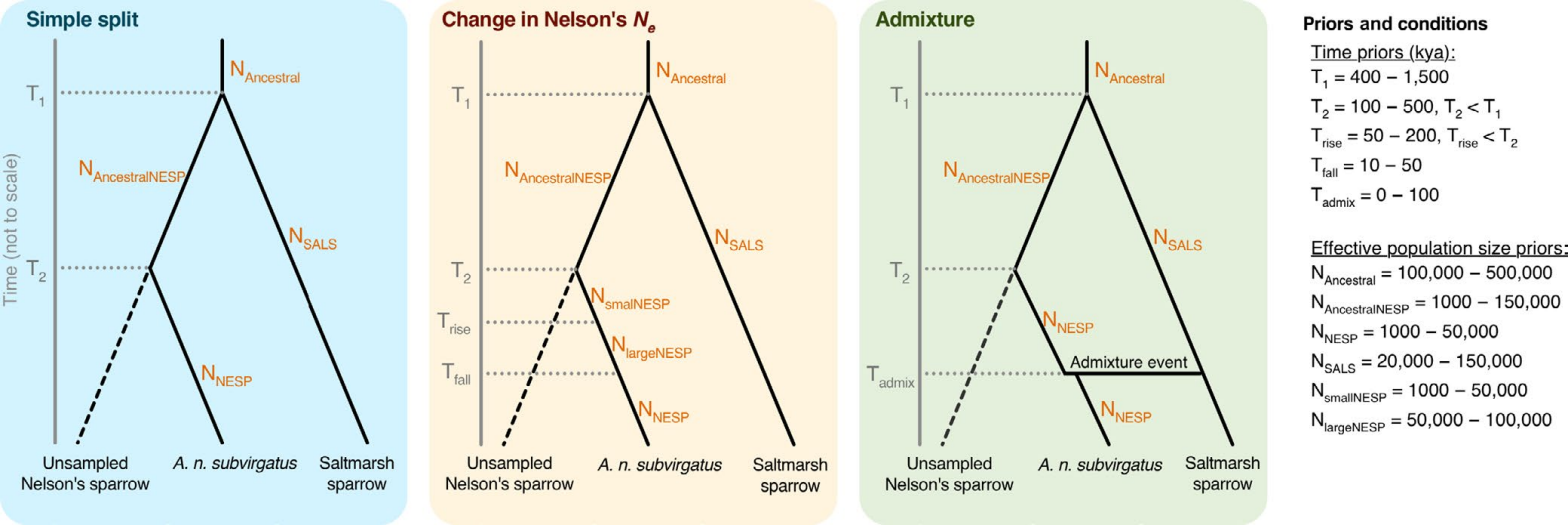
Demographic scenarios simulated in DIYABC. The time axis goes from the present, at the base of the axis, backward in time. Priors for the timing of events are labeled “*T*_x_.” Priors for effective population sizes of each branch are labeled “*N*_x_” on the corresponding branch. The effective population size of the unsampled Nelson's sparrow lineage (representing inland *A. n. nelsoni*) is not modeled. All priors were uniform priors

To investigate whether the changes in effective population size seen in the PSMC were real or due to admixture between the two species, we used approximate Bayesian computation, implemented in DIYABC v2.1.0 (Cornuet et al., 2014) to estimate the posterior probabilities of three different scenarios for the demographic history of the populations. DIYABC has been shown to have limited ability to discriminate between a large number of complex models (Cabrera & Palsbøll, 2017) so we limited our investigations to three, relatively simple scenarios (Figure 2). The scenarios that we tested were as follows: (a) a “simple split” scenario, similar to Greenlaw's (1993) hypothesis, where the ancestor to both species was subdivided into an inland, ancestral Nelson's population and a coastal, saltmarsh sparrow population, Nelson's sparrow population further splitting, giving rise to the inland *A. n. nelsoni* and the coastal, sampled *A. n. subvirgatu*s; (b) a “change in Nelson's Ne” scenario which follows the simple split scenario except, after diverging from *A. n. nelsoni*, *A. n. subvirgatus* goes through an increase and then a decrease in effective population size as seen in the PSMC plot (see Results); and finally (c) an “admixture” scenario which also follows the simple split model but after *A. n. subvirgatus* has diverged from *A. n. nelsoni*, an admixture event between *A. n. subvirgatus* and the saltmarsh sparrow creates a new, admixed lineage of *A. n. subvirgatus*. In all scenarios, *A. n. nelsoni* is a ghost lineage (Figure 2) since it is not sampled in this study.

Three sets of 2000 autosomal SNPs were selected, and the DIYABC simulations were repeated with each set to ensure results were robust to the subset of SNPs chosen. High‐quality SNPs were selected that had no missing data, had mean depth >8 and <12, were sequenced to a depth of at least five in all individuals (high‐confidence genotype calls are necessary for demographic history reconstruction), were within 1 standard deviation of the mean *F*_ST_ to ensure they were effectively neutral, were at least 20 kb apart to reduce linkage, and then chosen at random without replacement.

Uniform priors were used for all split times and effective population sizes. A generation length of 2.3 years was used to convert time in generations to time in years. In all scenarios, a wide prior of 400,000–1.5 million YA was used for the split between saltmarsh and Nelson's sparrows (*T*_1_) as estimates using mtDNA put the split at 600,000 YA (Rising & Avise, 1993) and mean *F*_ST_ is high between the species (see Section 3). A split of 100,000–500,000 YA was used for the divergence of *A. n. subvirgatus* from the ancestral Nelson's sparrow population (*T*_2_), which was set to occur after *T*_1_ (*T*_2_ < *T*_1_). Priors for the effective population size of saltmarsh sparrow (N_SALS_) and the sampled Nelson's sparrow subspecies (N_NESP_) were set to 20,000–150,000 and 1,000–50,000, respectively, based on their census population sizes (saltmarsh sparrow: 53,000, Wiest et al., 2016; *A. n. subvirgatus*: approximately 10,000, Wiest et al., 2016, Erskine, 1992, Rivard, Shaffer, & Falardeau, 2006) and the results of test simulations, which indicated that large priors were needed for these parameters. We had little information about the effective population size of the ancestral Nelson's sparrow (*N*_AncestralNESP_) so we used a wide prior of 1,000–150,000 individuals. PSMC indicated that the ancestor to saltmarsh and Nelson's sparrows (*N*_Ancestral_) may have had very large effective population size, so we used 100,000–500,000 as a prior range for this parameter.

In the “change in Nelson's *N*_e_” scenario, we used the PSMC plot to guide prior estimates for the rise and fall in Nelson's effective population size. The prior for the timing of the rise in Nelson's effective population size was set to 50,000–200,000 YA, and the subsequent fall in effective population size was set to 10,000–50,000 YA. Priors for the effective population sizes in this scenario were also based on the PSMC plot. We used 1,000–50,000 for the population size range prior to the increase in *N*_e_ (*N*_smallNESP_) and 50,000–100,000 for the effective population size when *N_e_* increased. In the “admixture” scenario, the timing of the admixture event between saltmarsh and Nelson's sparrow was set to occur within the last 100,000 years. Again, this prior range was guided by the PSMC plot for Nelson's sparrows. The prior for the admixture proportion was left as the default 0.001–0.999.

Out of a possible 16 summary statistics, we chose ten: the mean and variance of nonzero values, and proportion of zero values for the genic diversity of each species; the mean and variance of nonzero values for the pairwise *F*_ST_; and the mean and variance of nonzero values of Nei's pairwise distance. We ran 10^6^ simulations for each scenario (i.e., three million simulations, repeated three times for each set of 2,000 loci) and estimated the posterior probabilities of the scenarios using the logistic regression approach with the top 1% of simulated datasets. The posterior error rate for the scenario choice was estimated by simulating 1,000 pseudo‐observed datasets, taking the scenario ID and parameter values from the 500 simulated datasets closest to the observed data.

## 3 RESULTS

The final reference genome assembly generated by ALLPATHS‐LG consisted of 44,080 contigs with an N50 of 66.4 kb and 2,672 scaffolds with an N50 of 8.427 Mb. Contig length was 1.03 Gb, and the total scaffold length was 1.07 Gb. Based on a 1.0 Gb genome, sequencing coverage for the assembly was 83X. Statistics for the final assembly are included in Table S2. We assessed the completeness of our reference assembly by searching for a vertebrate set of 3,023 single‐copy orthologs using BUSCO version 1.2 (Simão et al., 2015). Our final saltmarsh sparrow reference genome contained a single and complete copy of 83.6% of the genes in the vertebrate set and a partial copy of an additional 7.5% of the genes in this set. We found 0.5% of the BUSCO vertebrate genes more than once within the reference genome, and 8.8% of the BUSCO genes were missing from the saltmarsh sparrow reference.

**Figure 3:**
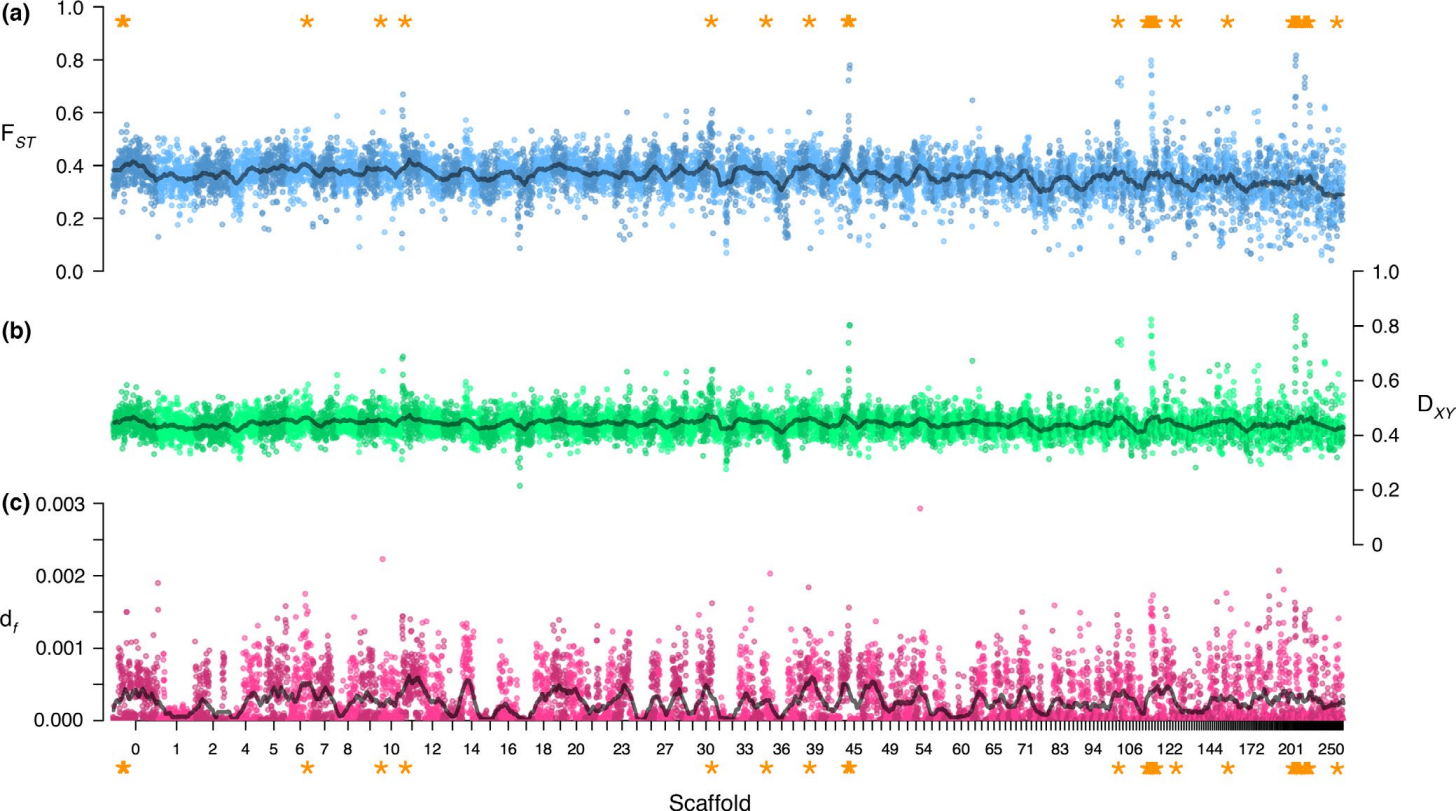
Genome‐wide patterns of divergence between saltmarsh and Nelson's sparrows. Panel a: genome‐wide estimates of *F*_ST_; Panel b: genome‐wide estimates of Dxy; Panel c: density of fixed SNPs across the genome. All results are averaged over 100‐kb windows and are presented for the largest scaffolds. Elevated windows (regions of the genome with both *F*_ST_ estimates and *df* greater than the 99th percentile of the empirical distribution) are marked with orange asterisks

Populations of saltmarsh and Nelson's sparrows exhibited high levels of genome‐wide divergence. Individual sparrows clustered strongly by species in a PCA based on 7.2 million SNPs, with PC axis one explaining 40.9% of the observed variation (Figure S1). Genome‐wide average *F*_ST_ (across all autosomal SNPs) was 0.32 (Figure 3a; Figure S2), indicating high baseline divergence between these two species. Genome‐wide estimates of *D*_xy_ showed a similar pattern to *F*_ST_ (Figure 3b). We identified 234,508 fixed SNPs between saltmarsh and Nelson's sparrows, which appear to be uniformly distributed across the genome (1.26% of which were in coding regions; Figure 3c).

**Figure 4:**
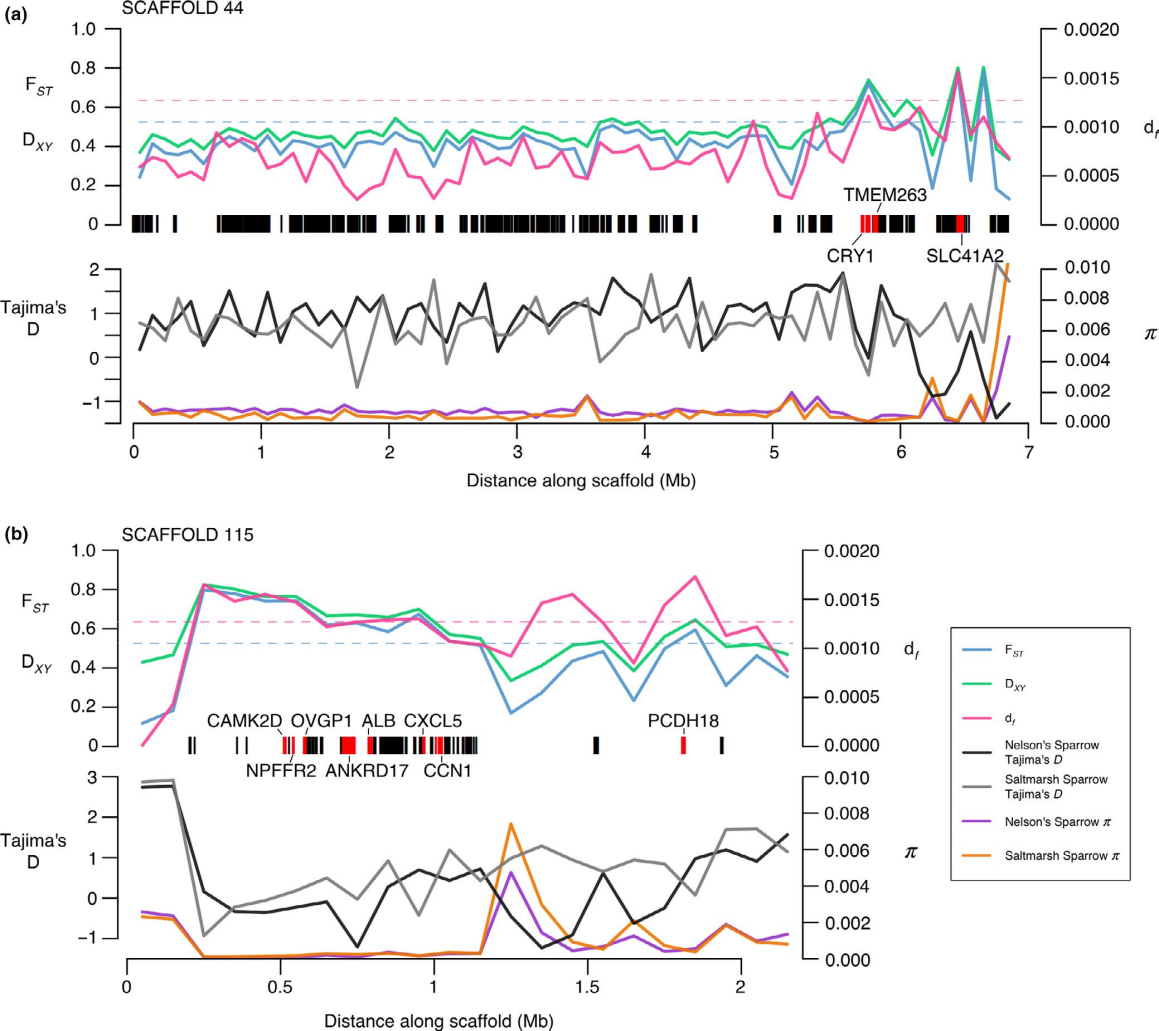
Windowed summary statistics along two scaffolds within regions of elevated divergence between saltmarsh and Nelson's sparrows. Divergence is summarized by *F*_ST_, *D*_XY,_ and *df* in 100‐kb windows in the upper panel for (a) scaffold 44 and (b) scaffold 115. The 99% thresholds for *F*_ST_ and *df* are shown by the blue and pink dotted lines, respectively. All annotated genes are shown by the black bars. Genes that fell within a region of elevated divergence and that we hypothesized to have functional significance within these species are highlighted in red. Nucleotide diversity (π) and Tajima's *D* are shown for each species and scaffold along the bottom of each plot

Based on our different approaches, we identified numerous regions of the genome that exhibited elevated levels of divergence (measured as either *F*_ST_ or the number of fixed SNPs in a window). We identified 90 windows across 39 scaffolds with *F*_ST_ estimates greater than the 99th percentile of the empirical distribution (Tables S3 and S4). We also identified 94 windows across 42 scaffolds exhibiting values of *df* greater than the 99th percentile of the overall distribution (Tables S5 and S6). When combining these two criteria, we found 33 windows across 16 scaffolds that showed elevated divergence using both *F*_ST_ estimates and the density of fixed SNPs (hereafter *elevated regions*, Table S7). When comparing elevated regions to the rest of the genome, we saw higher estimates of *F*_ST_, *D*_xy, _and *df* inside of the elevated regions (*F*_ST_ = 0.616; *D*_xy_ = 0.6795; *df = *0.0013) versus outside of the elevated regions (*F*_ST_ = 0.309; *D*_xy_ = 0.4412; *df = *0.00022; Table 2). Within these elevated regions, we identified 19 genes with putative roles in adaptive differences between the species (Table 1, Figure 4). Of these genes, nine were linked to osmoregulatory function, two were linked to reproductive differences between the species, two were linked to immune response, and one was linked to circadian rhythm. Enrichment analyses of these genes identified several pathways, including some linked to a priori hypotheses: intracellular calcium‐activated chloride channel activity (potentially linked to osmoregulatory function; *p* = .0067), regulation of circadian rhythm (potentially linked to nest initiation in relation to tidal cycles; *p* = .023), and sodium:potassium‐exchanging ATPase activity (potential link to osmoregulatory function; *p* = .023). Additional genes with potential tidal marsh or other adaptive functions were identified by either the *F*_ST_ or *df* criteria alone (Tables S3–S6). Nucleotide diversity was decreased within these elevated windows, although not statistically significantly given the high genome‐wide variation (Table 2). This could be evidence for selective sweeps in these regions. Lastly, based on a literature review and a priori predictions, we identified several a priori candidate genes linked to putative tidal marsh adaptations (Table 3). Of these candidates, only two had an *F*_ST_ value greater than the upper bound of the 95% confidence interval for genome‐wide *F*_ST_ (Table 3). These candidates included CRY1 (regulation of circadian rhythm: *F*_ST_ = 0.676, 86 fixed SNPs) and TYRP1 (melanin biosynthetic process: *F*_ST_ = 0.553, 44 fixed SNPs).

**Table 2:** Summary statistics for regions of the genome inside versus outside windows of elevated divergence

	Inside windows		Outside windows	
	Saltmarsh	Nelson's	Saltmarsh	Nelson's
D*xy*	0.6795 (0.0805)		0.4412 (0.0428)	
π	0.00029 (0.00017)	0.00044 (0.00043)	0.00101 (0.00162)	0.00130 (0.00153)
Tajima's *D*	0.34 (0.57)	0.36 (0.79)	0.33 (0.77)	0.81 (0.61)
*df*	0.0013 (0.0002)		0.00022 (0.00033)	
*F*_ST_	0.616 (0.087)		0.309 (0.070)	

Note

The mean (standard deviation) for each summary statistic is presented for the 37 windows of 100 kb in length which were above the upper 99th percentile of the *F*_ST_ and *df* distribution, along with the mean values for the rest of the autosomal genome.

**Table 3:** Genetic divergence measured within candidate gene regions (identified a priori)

Candidate Gene	Biological Function	Scaffold	Number of variable sites	Mean *F*_st_	Number of Fixed SNPs	Percentage of sites that are fixed
Bill morphology						
BMP4	Beak morphogenesis	185	223	0.307	1	0.0024
calm1^a^	Calcium ion binding	2	228	0.278	0	0
calm1^a^	Calcium ion binding	28	423	0.444	80	0.0812
NOG2L^b^	Binds BMP4					
GREM1^b^	Regulates BMP					
WISP3	Appears to be required for normal postnatal skeletal growth and cartilage homeostasis	21	484	0.375	37	0.0456
WNT1	Bone development, BMP signaling pathway	257	441	0.297	16	0.0377
WNT11	Bone mineralization	199	1,305	0.246	58	0.0445
WNT16^c^	Bone remodeling, keratinocyte differentiation, and proliferation	3	286	0.368	0	0
WNT3A	BMP signaling pathway	12	462	0.431	27	0.0482
WNT5A	Response to calcium ion, BMP signaling	18	187	0.347	0	0
Bill morphology and kidney function						
WNT4^a^	Kidney morphogenesis, regulation of bone mineralization,	184	173	0.431	27	0.0641
WNT4^a^	Kidney morphogenesis, regulation of bone mineralization,	91	294	0.342	26	0.0472
Kidney function						
MAPK13	Response to osmotic stress	101	207	0.473	10	0.0190
SLC6A1^a^	Sodium‐dependent transporters	16	386	0.380	18	0.0294
SLC6A1^a^	Sodium‐dependent transporters	104	534	0.310	9	0.0150
SLC6A2	Sodium‐dependent transporters	22	653	0.349	8	0.0063
SLC6A3^b^	Sodium‐dependent transporters					
SLC6A4^a^	Sodium‐dependent transporters	94	421	0.375	2	0.0041
SLC6A4^a^, ^c^	Sodium‐dependent transporters	41	362	0.324	6	0.0099
SLC6A5	Sodium‐dependent transporters	88	436	0.308	2	0.0029
SLC6A6	Sodium‐dependent transporters	18	224	0.435	29	0.0422
SLC6A7	Sodium‐dependent transporters	13	257	0.446	1	0.0019
SLC6A8	Sodium‐dependent transporters	18	160	0.414	2	0.0041
SLC6A9^a^	Sodium‐dependent transporters	24	609	0.355	1	0.0009
SLC6A9^a^	Sodium‐dependent transporters	52	242	0.399	32	0.0638
SLC6A10^b^	Sodium‐dependent transporters					
SLC6A11	Sodium‐dependent transporters	104	966	0.293	1	0.0008
SLC6A12	Sodium‐dependent transporters	16	433	0.433	11	0.0153
SLC6A13	Sodium‐dependent transporters	16	487	0.483	18	0.0257
SLC6A14	Sodium‐dependent transporters	38	428	0.298	27	0.0489
SLC6A15^c^	Sodium‐dependent transporters	3	415	0.410	4	0.0061
SLC6A16^b^	Sodium‐dependent transporters					
SLC6A17	Sodium‐dependent transporters	273	531	0.347	44	0.0747
SLC6A18^b^	Sodium‐dependent transporters					
SLC6A19	Sodium‐dependent transporters	27	508	0.375	2	0.0021
SLC6A20	Sodium‐dependent transporters	4	227	0.281	0	0
SLC8A1	Sodium calcium exchangers	23	286	0.460	70	0.1343
SLC8A2	Sodium calcium exchangers	338	376	0.277	22	0.0486
SLC8A3	Sodium calcium exchangers	17	142	0.432	1	0.0024
SLC34A1	Sodium phosphate cotransporters	13	260	0.395	4	0.0093
SLC34A2	Sodium phosphate cotransporters	9	217	0.440	36	0.0691
SLC34A3^b^	Sodium phosphate cotransporters					
SLC28A1	Sodium‐coupled nucleoside transporters	24	210	0.352	0	0
SLC28A2^b^	Sodium‐coupled nucleoside transporters					
SLC28A3	Sodium‐coupled nucleoside transporters	90	323	0.500	105	0.1342
SLC24A1	Sodium/calcium–potassium exchangers	84	217	0.458	5	0.0093
SLC24A2^b^	Sodium/Calcium‐Potassium exchangers					
SLC24A3^b^	Sodium/calcium–potassium exchangers					
SLC24A4^a^	Sodium/calcium–potassium exchangers	68	864	0.325	5	0.0033
SLC24A4^a^	Sodium/calcium–potassium exchangers	2	597	0.392	0	0
SLC24A5	Sodium/calcium–potassium exchangers	24	210	0.352	0	0
SLC24A6^b^	Sodium/calcium–potassium exchangers					
SLC23A1^a^	Sodium‐dependent ascorbic acid transporters	83	195	0.349	2	0.0033
SLC23A1^a^	Sodium‐dependent ascorbic acid transporters	152	239	0.377	8	0.0182
SLC23A2	Sodium‐dependent ascorbic acid transporters	240	583	0.343	17	0.0240
SLC23A3	Sodium‐dependent ascorbic acid transporters	0	175	0.359	9	0.0210
SLC23A4^b^	Sodium‐dependent ascorbic acid transporters					
SLC20A1	Type III sodium phosphate cotransporters	262	288	0.217	22	0.0460
SLC20A2	Type III sodium phosphate cotransporters	7	301	0.412	44	0.0621
SLC13A1^c^	Sodium sulfate/carboxylate cotransporters	3	330	0.392	3	0.0049
SLC13A2^c^	Sodium sulfate/carboxylate cotransporters	41	298	0.421	7	0.0135
SLC13A3	Sodium sulfate/carboxylate cotransporters	35	386	0.337	2	0.0030
SLC13A4	Sodium sulfate/carboxylate cotransporters	16	463	0.400	60	0.0891
SLC13A5	Sodium sulfate/carboxylate cotransporters	373	60	0.536	0	0
SLC10A1	Sodium bile salt cotransporters	17	165	0.392	1	0.0023
SLC10A2	Sodium bile salt cotransporters	1	285	0.371	1	0.0021
SLC10A3^b^	Sodium bile salt cotransporters					
SLC10A4	Sodium bile salt cotransporters	100	219	0.533	62	0.1442
SLC10A5^b^	Sodium bile salt cotransporters					
SLC10A6^b^	Sodium bile salt cotransporters					
SLC10A7^b^	Sodium bile salt cotransporters					
Melanic plumage						
SLC45A2	Melanin biosynthetic process	53	202	0.379	5	0.0090
MC1R	Melanin biosynthetic process, pigmentation	103	151	0.418	28	0.0684
TYRP1^c^	Melanin biosynthetic process	81	185	0.553	44	0.0879
Nest initiation timing						
CRY1^a^	Regulation of circadian rhythm	44	169	0.676	86	0.1137
CRY1^a^	Regulation of circadian rhythm	123	504	0.241	41	0.0724
CRY2	Regulation of circadian rhythm	17	245	0.330	0	0
NPAS2^a^	Regulation of circadian rhythm	1	443	0.404	8	0.0087
NPAS2^a^	Regulation of circadian rhythm	38	358	0.446	56	0.0690
CIPC	Regulation of circadian rhythm	2	135	0.398	2	0.0045
CLOCK	Regulation of circadian rhythm	66	460	0.410	7	0.0071
MAPK10^a^	Regulation of circadian rhythm	30	184	0.301	0	0
MAPK10^a^	Regulation of circadian rhythm	30	206	0.382	14	0.0316
MAPK8	Regulation of circadian rhythm	10	320	0.432	0	0.0000
MAPK9	Regulation of circadian rhythm	13	258	0.345	3	0.0054
PER1^b^	Regulation of circadian rhythm					
PER2	Regulation of circadian rhythm	89	503	0.295	0	0
PER3	Regulation of circadian rhythm	86	322	0.388	3	0.0052
			MEAN	0.383	MEAN	0.0288

Note

The table includes the mean *F*_ST_ and number of fixed SNPs found between Nelson's and saltmarsh sparrows within the bounds of the coding region of each gene, plus 20 kb upstream and downstream. The percentage of sites that are fixed is calculated as the percentage of all sites in the region. Genes highlighted in gray have *F*_ST_ greater than the upper bound of the 95% confidence interval.

a Duplicated annotation within the reference genome.

b Not annotated in the saltmarsh sparrow reference genome.

c Putatively sex linked.

**Figure 5:**
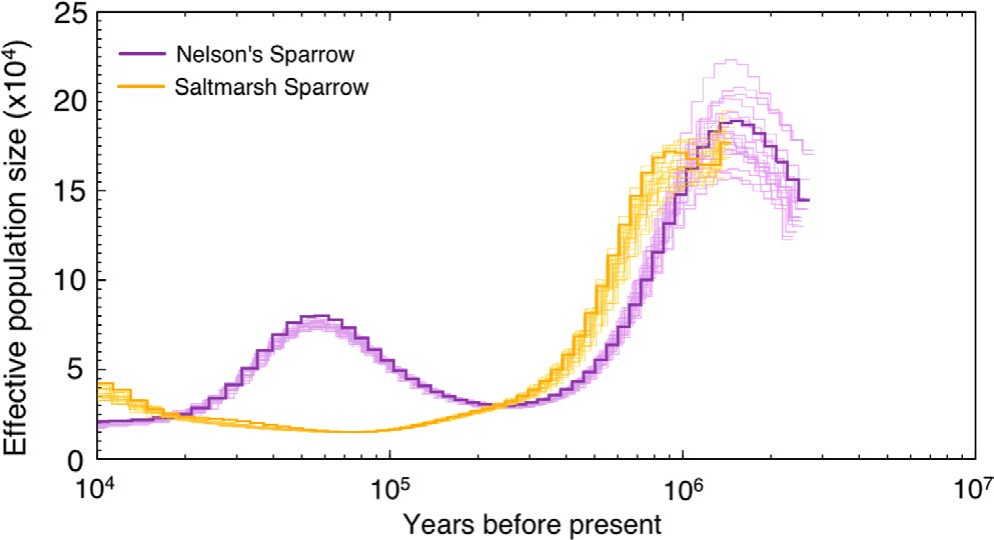
Demographic history inferred using PSMC. We performed 100 bootstrap replicates (thin lines) for each species. Saltmarsh sparrows (orange) appear to have had a long history of relatively small population size since they diverged from the common ancestor, which had much larger effective population size. Nelson's sparrows (purple) have a more complex history, with either a change in population size or a change in population structure occurring approximately 50–100 kya

Analyses of population size history with PSMC revealed that both species diverged from an ancestral population that had much larger effective size (150,000–200,000) than either extant species (Figure 5). The saltmarsh sparrow appears to have had a long history of relatively constant, small effective size of approximately 30,000–50,000 since divergence from the common ancestor, while Nelson's sparrow has had a more complex history. A hump in Nelson's sparrow PSMC plot at roughly 50,000–100,000 YA indicates that either an increase in effective population size or a change in population structure, such as an admixture event, occurred at this time, followed by a decrease in population size to approximately 20,000 individuals 10,000 YA. Our DIYABC analysis confirmed that the hump in the PSMC plot was likely an admixture event. The “admixture scenario” was highly favored (posterior probability = 1.000) over the “change in Nelson's Ne” scenario (posterior probability = 0) and the “simple split” scenario (posterior probability = 0). Confidence in scenario choice was high (posterior predictive error of scenario choice = 0.000), and this was true for all three sets of 2000 SNPs that were used. However, the timing of the admixture event differed for the DIYABC analysis, which estimated the event to have occurred within the last 10,000 years (Table 4), rather than 50,000–100,000 YA as indicated in the PSMC plot.

**Table 4:** DIYABC parameter estimates from the admixture model using each of the three SNP subsets

	Divergence time (*T*_1_)	Timing of admixture (*T*_admix_)	Ancestral effective population size (*N*_Ancestral_)	Saltmarsh effective population size (*N*_SALS_)	Nelson's effective population size (*N*_NESP_)
SNP set 1	552,000 (384,100–1,232,800)	7,268 (1,649–19,734)	414,000^a^ (155,000–497,000)	83,200 (34,900–134,000)	29,400 (7,850–48,400)
SNP set 2	655,500 (402,500–1,281,100)	5,382 (1,233–14,674)	316,000 (121,000–488,000)	109,000 (51,800–145,000)	20,400 (4,320–45,600)
SNP set 3	478,400^b^ (377,200–1,099,400)	5,474 (1,180–15,157)	444,000^a^ (196,000–498,000	138,000^a^ (93,900–149,000)	21,100 (4,780–46,000)

Note

For each parameter, the median of the posterior distribution is given with the 95% HPD in parentheses.

a May not be well estimated from this SNP subset, as the mean was approaching the upper bound of the prior.

b May not be well estimated from this SNP subset, as the mean was approaching the lower bound of the prior.

The estimates of time since divergence of the saltmarsh and Nelson's sparrow lineages were similar for the two analyses, with PSMC suggesting divergence at ~1 million YA and the three median estimates from DIYABC ranging 478,400–655,500 YA (95% HPD range of 377,200–1,281,100 YA; Table 4). Note that the lowest estimate stemming from the 3rd SNP subset may not be robust, given that the mean was approaching the lower bound of the prior. The DIYABC parameter estimates for the saltmarsh sparrow, Nelson's sparrow, and ancestral Ne were relatively consistent across the three SNP subsets (Table 4) and largely consistent with the estimates from PSMC, with Nelson's sparrow having a smaller population size than saltmarsh sparrow, although note that DIYABC estimates are averaged across the full time span of the analysis, rather than for a particular time period, as for PSMC. Median saltmarsh sparrow Ne averaged 110,000 (95% HPD range 34,900–149,000) and median Nelson's sparrow averaged 23,600 (95% HPD range 4320–48,400) across the three SNP subset analyses. Ancestral Ne estimates averaged 391,000 (95% HPD range 121,00–490,000). One of the SNP subsets for saltmarsh sparrow Ne and two of the SNP subsets for ancestral Ne produced means that were approaching the upper bound of the prior.

## 4 DISCUSSION

Whole‐genome comparisons of saltmarsh and Nelson's sparrows revealed high baseline divergence between species (genome‐wide *F*_ST_ = 0.32). We identified a high density of fixed SNPs (~234,000), which appear to be uniformly distributed across the genome. These patterns share commonalities with that observed in the collared (*Ficedula albicollis*) and pied (*F. hypoleuca*) flycatcher, which exhibit high baseline divergence in allopatry (*F*_ST_ = 0.357; Ellegren et al., 2012), a large number of fixed SNPs (239,745), low rates of heterospecific pairings, and fitness reductions in hybrids (Svedin, Wiley, Veen, Gustafsson, & Qvarnström, 2008). The latter two observations have also been made in saltmarsh and Nelson's sparrows (Maxwell, 2018; Walsh, Maxwell, & Kovach, 2018; Walsh et al., 2016). Conversely, flycatchers may exhibit a deeper divergence time (<2 million years; Ellegren et al., 2012) than that estimated for saltmarsh and Nelson's sparrows—500,000–1 million years based on our DIYABC and PSMC analyses in this study, consistent with 600,000 years based on mitochondrial differentiation (Rising & Avise, 1993). Our findings suggest higher genome‐wide patterns of divergence than between other shallowly diverged, hybridizing taxa. For instance, Poelstra et al. (2014) and Toews et al. (2016) found only a small number of elevated regions and a small number of fixed SNPs across the entire genome (82 and 74, between carrion and hooded crows—*Corvus corone and C. cornix*—and golden‐winged and blue‐winged warblers—*Vermivora chrysoptera and V. cyanoptera*—respectively). Taken together, the high levels of divergence observed in these congeneric *Ammodramus* sister species may shed light on how both selective and demographic processes shape patterns of genetic differentiation.

Demographic processes, including a complex history of splitting, colonization events, and secondary contact, with potentially multiple past admixture events, coupled with limited contemporary gene flow, are likely important factors in shaping the high baseline divergence we observed between saltmarsh and Nelson's sparrows. It is hypothesized that the ancestral population was split by Pleistocene glaciation, resulting in an isolated interior population (Nelson's sparrows) and a coastal refugia population (saltmarsh sparrows); our results confirm prior work dating the time of this split at roughly half a million to one million years ago. Following differentiation, Nelson's sparrow populations then spread eastward making secondary contact with coastal saltmarsh sparrow populations (Greenlaw, 1993). Given repeating patterns of glaciation and retreat, it is possible that populations expanded and retracted into refugia on more than one occasion. Indeed, the differing results of our PSMC and DIYABC analyses with respect to the timing of admixture suggest that there may have been at least two distinct admixture events—one 50,000–100,000 years ago (supported by PSMC) and a more recent event after the last glacial retreat, within the last 10,000 years (supported by DIYABC). These results may reflect different limitations of the two approaches—PSMC is known to lack sensitivity for detecting demographic processes that occurred within the relatively recent past (Nadachowska‐Brzyska et al., 2016) and DIYABC, accordingly, may have been more prominently influenced by recent events. Population size estimates from both approaches support long periods of time with relatively small population sizes and declines relative to the ancestral population. Thus, a history of reduction in population sizes (via glacial retreat, splitting, or founder events when re‐colonizing tidal marsh habitats) may have provided a prominent role for drift in shaping genomic divergence between these taxa.

In addition to historical processes, patterns of contemporary gene flow may also shape the genomic landscape. Despite a 200‐km hybrid zone between saltmarsh and Nelson's sparrows (Hodgman, Shriver, & Vickery, 2002; Shriver et al., 2005), our sampled individuals are predominantly from allopatric populations separated by both geographic distance and a habitat gradient that may pose a selective barrier in this system (Greenlaw, 1993; Walsh et al., 2015). Divergence via drift in allopatry therefore may have progressed unfettered from potential homogenizing effects of gene flow, resulting in the observed patterns of high divergence across the genomic landscape (sensu Feder, Egan, & Nosil, 2012). Support for this idea comes from a study comparing whole genomes of saltmarsh and Nelson's sparrows from allopatric and sympatric populations (Walsh, Kovach, Olsen, Shriver, & Lovette, 2018). That study found that only about 5% of the fixed differences found in allopatry are present in sympatric populations, suggesting that when populations co‐occur contemporary gene flow homogenizes all but a small portion of the genomic landscape, which likely comprises important barrier loci (loci important in reproductive isolation; Feder et al., 2012; Nosil & Feder, 2012). Thus, drift—related to both historical and contemporary processes—seems to be an important factor in shaping the landscape of genomic divergence we have observed in these *Ammospiza* sparrows.

Despite a relatively high baseline of genome‐wide divergence between saltmarsh and Nelson's sparrows, we detected putative candidate regions of adaptation housed within elevated regions of differentiation (expressed both as elevated estimates of *F*_ST_ and as concentrated densities of fixed SNPs). This suggests that demographic processes alone are not responsible for the observed genomic landscape and supports a role for divergent selection in shaping the observed patterns of genome‐wide divergence between these taxa. Agreement on whether ecological divergence should be manifested in localized versus genome‐wide differentiation is generally lacking, and it is likely that different processes are operating at different stages of the speciation continuum (Feder et al., 2012; Hemmer‐Hansen et al., 2013; Via, 2012). Genomic islands of divergence—discrete regions of the genome with elevated divergence harboring clusters of loci underlying ecological adaptations—have been found in ecotypes or sister species (e.g., Hohenlohe, Bassham, Currey, & Cresko, 2012; Jones et al., 2012; Larson et al., 2017; Nosil, Harmon, & Seehausen, 2009). These islands are commonly thought to arise through divergence hitchhiking, whereby strong selection in conjunction with reduced recombination (due to linkage disequilibrium) results in coordinated evolution of multiple genes in the same genome region (Via, 2012), although other mechanisms, including regions of low genetic diversity, may also explain their presence (Cruickshank & Hahn, 2014). The prevalence, functional significance, and underlying mechanisms of these islands of divergence are not yet fully understood (Cruickshank & Hahn, 2014; Larson et al., 2017), and adaptive loci have also been found to be distributed more evenly across the genome (Strasburg et al., 2012). The pattern of high background differentiation coupled with peaks of elevated divergence distributed throughout the genomes of saltmarsh and Nelson's sparrows is consistent with expectations for populations that diverged in allopatry, in contrast to the clustered patterns of divergence found in species that diverged with ongoing gene flow (reviewed in Harrison & Larson, 2016). Under a scenario of ecological speciation, the differentiated genes within these elevated genomic regions underlie adaptations to the differential selective pressures faced by saltmarsh and Nelson's sparrows in allopatry. We hypothesize that several of the observed genetic differences represent ecologically favored alleles in either saline or freshwater habitats, alleles underlying adaptive behaviors to tidal versus nontidal environments, or alleles that represent other ecological differences between the species.

Using a multistep approach, we identified strong candidate regions for tidal marsh adaptations between saltmarsh and Nelson's sparrows. We identified several candidates with elevated *F*_ST_ estimates or numbers of fixed differences, with our most compelling candidates exhibiting elevated divergence at both of these metrics. Candidates with putative adaptive roles in tidal marshes include genes linked to osmoregulatory function, circadian rhythm, and melanin pigmentation. The genes SLC41A2 and ALB, for example, exhibited elevated *F*_ST_ estimates and a high density of fixed SNPs, and have roles in solute transport pathways that may occur in osmoregulatory function. SLC41A2 is a membrane Mg^2+^ transporter. Mg^2+^ is the second most abundant cation in seawater, with SLC41A2 identified as a Na^+^/Mg^2+^ exchanger that is highly expressed in the kidney of saltwater acclimated puffer fish (*Takifuga rubripes)* compared with closely related freshwater species (*T. obscurus*; Islam, Kato, Romero, & Hirose, 2011). SLC41A2 has also been previously found to exhibit reduced introgression and increased selection across the saltmarsh–Nelson's sparrow hybrid zone (Walsh et al., 2016), offering further support for an adaptive role for this gene. ALB is linked to the regulation of osmotic pressure of blood plasma and is a gene that was found to be under positive selection in the saline tolerant crab‐eating frog (*Fejervarya cancrivora*) compared with a morphologically similar, saline‐intolerant sister species (Shao et al., 2015).

Another candidate gene linked to tidal marsh adaptation identified in this study was CRY1, which is an important component of the circadian clock. In salt marshes, flooding affects nests during the highest spring tides; during this time, the entire marsh is flooded and nests can be inundated with water for an hour or two (Gjerdrum, Sullivan‐Wiley, King, Rubega, & Elphick, 2008). Monthly tidal flooding, therefore, is the leading cause of nest failure and an important driver of overall population trajectories for tidal marsh sparrows (Greenlaw & Rising, 1994; Shriver et al., 2007); synchronizing the 23‐ to 26‐day nesting cycle with the approximate 28‐day monthly tidal cycle is critical for individual fitness. Saltmarsh sparrows have greater nesting synchrony with tidal cycles compared with Nelson's sparrows, and this synchrony is associated with increased nesting success (Shriver et al., 2007; Walsh et al., 2016). Biological clocks are important for ensuring that the passage of time is synchronized with periodic environmental events (Kumar et al., 2010); as such, it is reasonable to hypothesize that CRY1 may play a role in the divergent nesting synchrony observed between these species. Expression patterns of CRY1 were shown to fluctuate with lunar periodicity in a lunar‐synchronized spawner, the golden‐lined spinefoot (*Siganus guttatus*; Ikegami, Takeuchi, Hur, & Takemura, 2014), supporting a link between CRY1 and lunar cycles.

The final putative candidate for tidal marsh adaptation that we identified was TYRP1, which is an important component of the melanin biosynthesis pathway and has been found to play an important role in determining plumage color in birds (Bourgeois et al., 2016; Minvielle, Cecchi, Passamonti, Gourichon, & Renieri, 2009; Xu, Zhang, & Pang, 2013). This finding supports previous work identifying SLC45A2 (another gene associated with plumage melanism) to be under divergent selection in this system (Walsh et al., 2016). Saltmarsh and Nelson's sparrows have subtle plumage differences—with saltmarsh sparrows showing darker breast and flank streaking and face coloration than Nelson's sparrows (Shriver et al., 2007)—consistent with phenotypic patterns observed in other vertebrates spanning tidal marsh gradients (Grinnell, 1913; Luttrell et al., 2014). Tidal marsh taxa are grayer or blacker than their upland relatives, due to a greater expression of eumelanin relative to pheomelanin (Greenberg & Droege, 1990). This salt marsh melanism is thought to confer adaptive benefits to tidal marsh birds either via enhanced predator avoidance (from background matching with the gray‐black marsh mud; Greenberg & Droege, 1990) or resistance to feather degradation (increased melanin concentrations slow degradation rates by salt‐tolerant feather bacteria; Olsen, Greenberg, Liu, Felch, & Walters, 2009; Peele, Burtt, Schroeder, & Greenberg, 2009).

In addition to candidate genes that were in line with our a priori predictions for tidal marsh adaptations, we also identified several genes under selection that are related to spermatogenesis. Differences in mating strategies in saltmarsh and Nelson's sparrows (Greenlaw, 1993) coupled with strong assortative mating in the hybrid zone (Walsh et al. in press) support a role for premating barriers, which could include sperm competition or incompatibilities between the species. These candidate regions, coupled with the osmoregulatory and circadian rhythm candidates described above, will provide directions for future research in this system, including candidate gene work in more individuals across habitat types. Further, the full suite of genes identified by either *F*_ST_ or *df* criteria alone (Tables S3–S6) provides additional putative candidates for further investigation.

Our results demonstrate the effects of both ecological divergence and drift in driving high baseline levels of differentiation between two closely related sister taxa. The candidate genes for osmoregulatory response, circadian rhythm, and melanistic plumage that we identified in this study likely represent important lineage‐specific adaptations and add to a body of empirical evidence describing underlying mechanisms driving adaptation to harsh environments (Tong, Tian, & Zhao, 2017; Wu et al., 2014; Yang et al., 2016). Our findings, in particular, contribute to a growing list of candidate genes linked to salt tolerance and osmoregulation (Ferchaud et al., 2014; Gibbons, Metzger, Healy, & Schulte, 2017; Kahle, Rinehart, & Lifton, 2010; Walsh et al., 2019), suggesting that modifications to several pathways can result in adaptations to saline environments. Demographic analyses underscore the influence of population history, including multiple admixture events and population declines on the genomic landscape. These analyses also highlight that although the two species have persisted with relatively small effective population sizes since their initial divergence from a common ancestor, those population sizes were at least an order of magnitude higher than those of saltmarsh and Nelson's sparrows today (Wiest et al., 2016). High genome‐wide divergence and a high proportion of fixed SNPs distributed across the genomes of saltmarsh and Nelson's sparrows offer new perspectives into processes shaping the genomic landscape and offer empirical evidence for the shared roles of ecological divergence and demography in shaping evolutionary processes and genetic variation between these taxa.

## CONFLICT OF INTEREST

We have no competing interests.

## AUTHOR CONTRIBUTIONS

A.I.K. conceived and designed this study, with input from J.W., G.V.C., and M.D.M. J.W. and A.I.K. collected samples. J.W. and G.V.C. analyzed the data. J.W., G.V.C., and A.I.K. wrote the manuscript. M.D.M. provided guidance with bioinformatics analyses. W.K.T. led the transcriptome development and genome resequencing process. All authors read and approved the final manuscript.

## Data Availability

Our SNP dataset and the saltmarsh sparrow reference genome are available on the Dryad Data Repository (https://doi.org/10.5061/dryad.54gb2m9).
