# Supplementary material for: Divergent selection and drift shape the genomes of two avian sister species spanning a saline–freshwater ecotone
Source: Ecol Evol. 2019 Nov 7;9(23):13477–94. doi: 10.1002/ece3.5804 (PMC6912898; doi:10.1002/ece3.5804)
Supplement: Supplementary file 1 [file ECE3-9-13477-s001.docx]

**Divergent selection and drift shape the genomes of two avian sister species spanning a saline-freshwater ecotone**

**Jennifer Walsh^1,2,3†^, Gemma V. Clucas^1†^, Matthew D. MacManes^4,5^, Adrienne I. Kovach^1*^**

**Supporting Information:**

**Figure S1:** PCA plot generated using 6.3 million autosomal SNPs shows clear differentiation between saltmarsh and Nelson’s sparrows.


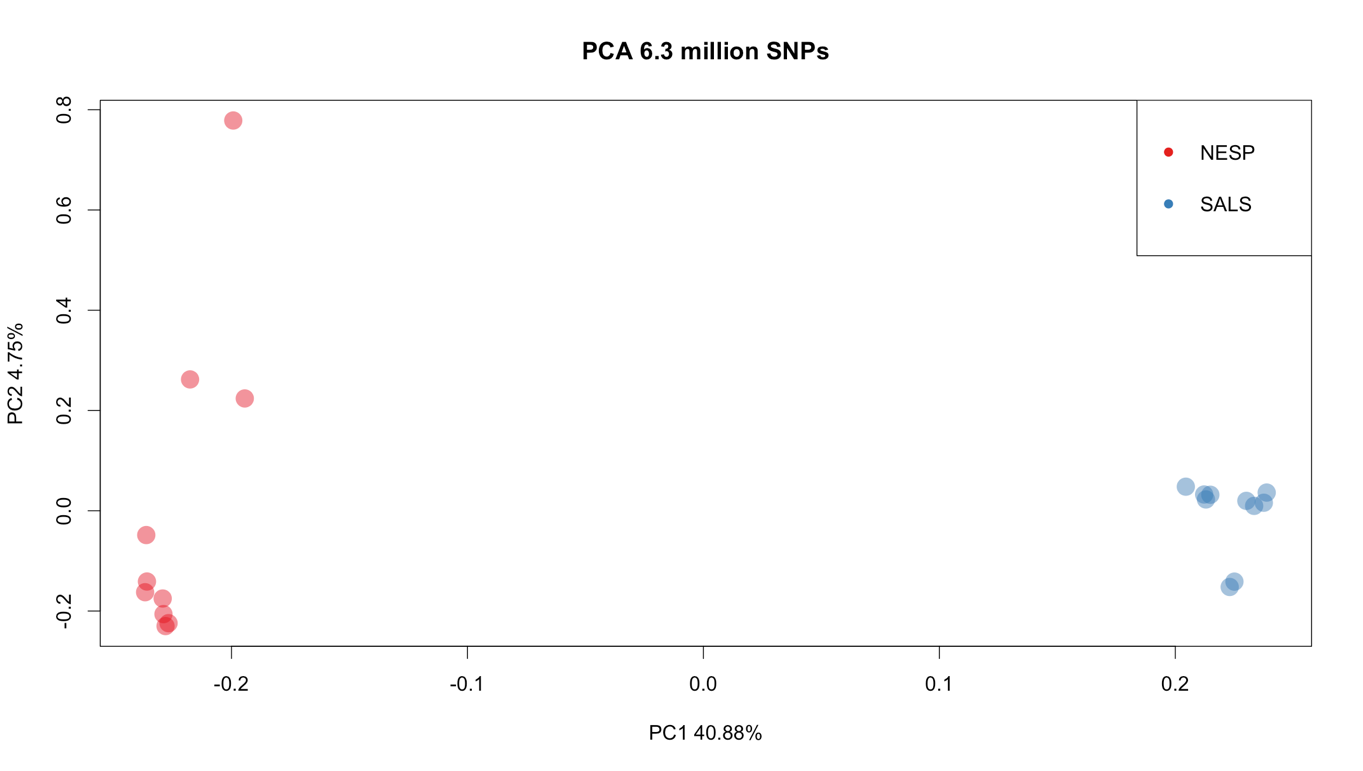


**Figure S2:** Histogram of autosomal F*_ST_* values between allopatric populations of saltmarsh and Nelson’s sparrows.


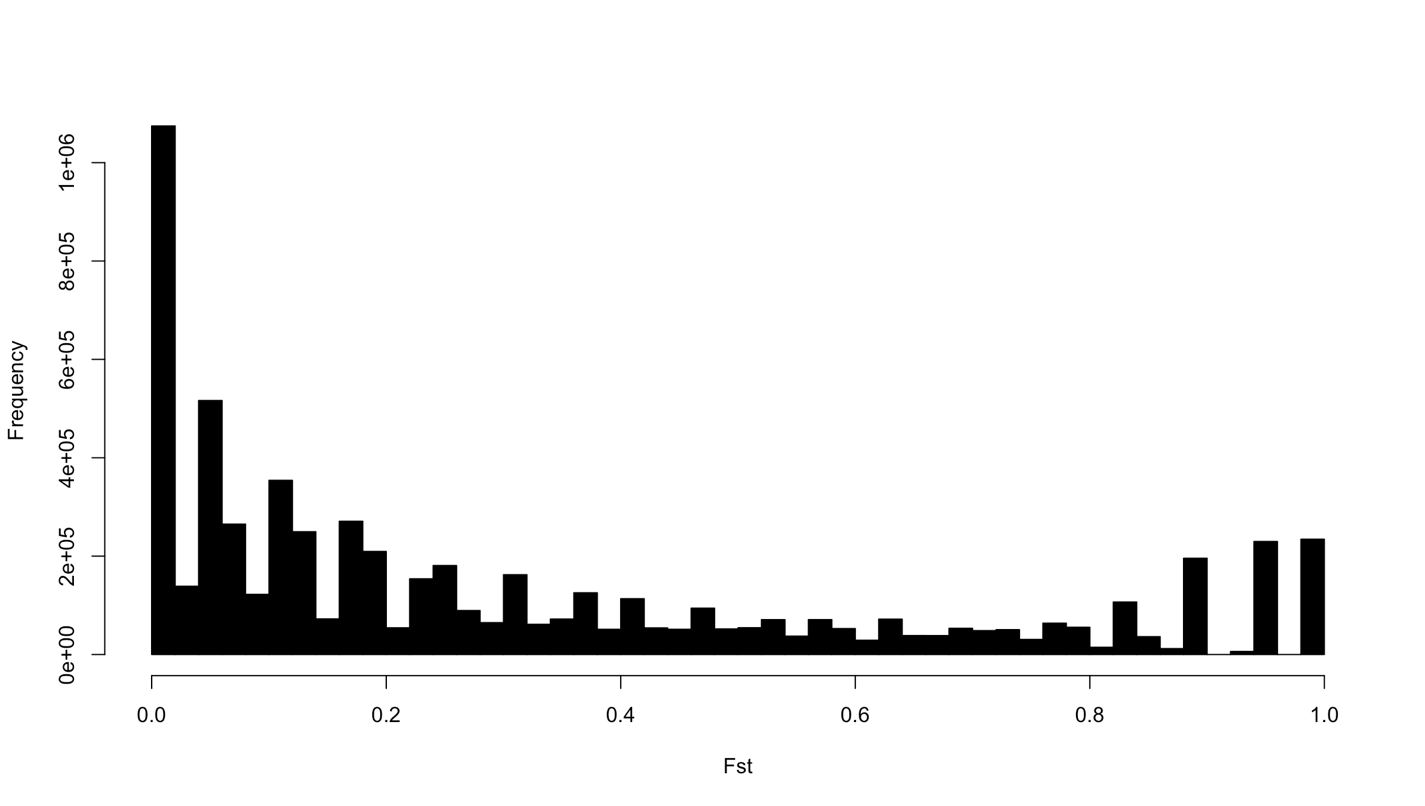


**Table S1:** Sample information and sampling locations for 20 saltmarsh and Nelson’s sparrows used for whole-genome re-sequencing. Table includes individual sample ID’s, species, sampling location, population type, sequencing statistics, and geographic coordinates.

| **Individual ID** | **Species** | **Summary** | **Sampling Location** | **Population Type** | **% of Paired Reads Mapped to Reference** | **Average Sequencing Coverage** | **Latitude** | **Longitude** |
| --- | --- | --- | --- | --- | --- | --- | --- | --- |
| LUB1 | Nelson's Sparrow | Re-sequenced Individual | Lubec, Maine | Allopatric | 98.0 | 6.7 | 44.822 | -66.991 |
| LUB2 | Nelson's Sparrow | Re-sequenced Individual | Lubec, Maine | Allopatric | 98.0 | 6.7 | 44.822 | -66.991 |
| LUB3 | Nelson's Sparrow | Re-sequenced Individual | Lubec, Maine | Allopatric | 98.2 | 6.5 | 44.822 | -66.991 |
| NAR1 | Nelson's Sparrow | Re-sequenced Individual | Narraguagus River - Maine | Allopatric | 97.3 | 5.0 | 44.551 | -68.891 |
| NAR2 | Nelson's Sparrow | Re-sequenced Individual | Narraguagus River - Maine | Allopatric | 98.0 | 6.3 | 44.551 | -68.891 |
| NAR3 | Nelson's Sparrow | Re-sequenced Individual | Narraguagus River - Maine | Allopatric | 98.0 | 6.1 | 44.551 | -68.891 |
| PRN1 | Nelson's Sparrow | Re-sequenced Individual | Penobscot, Maine | Allopatric | 97.5 | 5.2 | 44.591 | -68.859 |
| PRN2 | Nelson's Sparrow | Re-sequenced Individual | Penobscot, Maine | Allopatric | 98.1 | 5.8 | 44.591 | -68.859 |
| PRN3 | Nelson's Sparrow | Re-sequenced Individual | Penobscot, Maine | Allopatric | 98.1 | 5.7 | 44.591 | -68.859 |
| 2071-1 | Nelson's Sparrow | Re-sequenced Individual | Penobscot, Maine | Allopatric | 96.9 | 34.0 | 44.591 | -68.859 |
| FSM | Saltmarsh Sparrow | Re-sequenced Individual | Four Sparrow Marsh - New York City | Allopatric | 98.1 | 7.1 | 40.601 | -73.905 |
| IDL | Saltmarsh Sparrow | Re-sequenced Individual | Idlewild Marsh - New York City | Allopatric | 97.6 | 6.7 | 40.648 | -73.745 |
| IP | Saltmarsh Sparrow | Re-sequenced Individual | Ipswich, Massachusetts | Allopatric | 98.1 | 6.2 | 42.679 | -70.773 |
| MON | Saltmarsh Sparrow | Re-sequenced Individual | Momomoy Island, Massachusetts | Allopatric | 98.0 | 6.3 | 41.603 | -69.987 |
| 1914 | Saltmarsh Sparrow | Re-sequenced Individual | Sachuest Point - Rhode Island | Allopatric | 95.0 | 18.7 | 41.487 | -71.248 |
| 2071-2 | Saltmarsh Sparrow | Re-sequenced Individual | East River - Connecticut | Allopatric | 97.2 | 5.9 | 41.275 | -72.661 |
| 2071-3 | Saltmarsh Sparrow | Re-sequenced Individual | Shirley, New York | Allopatric | 97.2 | 6.7 | 40.770 | -72.893 |
| 2071-4 | Saltmarsh Sparrow | Re-sequenced Individual | Chapman's Landing - New Hampshire | Sympatric | 97.3 | 6.8 | 43.044 | -70.915 |
| 2071-5 | Saltmarsh Sparrow | Re-sequenced Individual | Parker River - Massachusetts | Sympatric | 97.2 | 5.4 | 42.773 | -70.819 |
| 2071-6 | Saltmarsh Sparrow | Re-sequenced Individual | Scarborough, Maine | Sympatric | 97.3 | 8.0 | 43.567 | -70.344 |
| 17545 | Saltmarsh Sparrow | Reference Genome | Marine Nature Center - Oceanside, New York | Allopatric | NA | NA | 40.621 | -73.622 |

**Table S2:** Assembly statistics for a male Saltmarsh Sparrow reference genome sampled from Oceanside, New York. Assembly was completed using ALL-PATHS-LG

| **Assembly Statistics:** |  |
| --- | --- |
| Total number of original fragment reads | 534444368 |
| Mean length of original fragment reads in bases | 101 |
| % GC content | 46.7 |
| Estimated genome size in bases | 1030158484 |
|  |  |
| Contig minimum size for reporting | 1000 |
| Number of contigs | 44080 |
| Number of contigs per Mb | 41 |
| Number of scaffolds | 2672 |
| Total contig length | 1036896301 |
| Total scaffold length, with gaps | 1074601438 |
| N50 contig size in kb | 66.4 |
| N50 scaffold size in kb | 8427 |
| N50 scaffold size in kb, with gaps | 8061 |
| Number of scaffolds per Mb | 2.49 |
| median size of gaps in scaffolds | 260 |
| median dev of gaps in scaffolds | 30 |
| ambiguities per 10,0000 bases | 11.22 |

**Table S3:** Windows exhibiting elevated divergence between saltmarsh and Nelson’s sparrows, assessed as regions with F*_ST_* estimates greater than the 99^th^ percentile of the empirical distribution. Table includes information on the region exhibiting elevated F*_ST_* estimates (scaffold and window start position), the number of SNPs contained within each window, mean F*_ST_*, and MAKER annotations for genes contained within 50 kb of the windows. Genes that have a hypothesized role in bill morphology are highlighted in red, those that have a hypothesized role in tidal marsh adaptations are highlighted in green, genes with hypothesized roles in circadian rhythm regulation are highlighted in blue, and genes with other potential adaptive functions are in grey.

| **Scaffold** | **Window Starting Position** | **Number of Variants in Window** | **Mean Fst** | **Maker Annotation** |
| --- | --- | --- | --- | --- |
| scaffold_0 | 6,200,001 | 432 | 0.525967 | STEAP3 |
|  |  |  |  | DBI |
|  |  |  |  | TMEM37 |
|  |  |  |  | SCTR |
|  |  |  |  | CFAP221 |
|  |  |  |  | ESYT3 |
|  |  |  |  | CEP70 |
|  |  |  |  | Faim |
|  |  |  |  | PARP9 |
|  |  |  |  | DTX3L |
|  |  |  |  | PARP14 |
| scaffold_0 | 9,800,001 | 522 | 0.526282 | No genes predicted in this region |
| scaffold_0 | 10,000,001 | 506 | 0.553127 | No genes predicted in this region |
| scaffold_10 | 1,300,001 | 657 | 0.602402 | ANXA8 |
|  |  |  |  | NPY4R |
|  |  |  |  | MSMB |
|  |  |  |  | WASHC2C |
|  |  |  |  | Zfand4 |
| scaffold_102 | 1,400,001 | 361 | 0.557735 | LDHB |
|  |  |  |  | Kcnj8 |
|  |  |  |  | ABCC9 |
|  |  |  |  | Cmas |
|  |  |  |  | Sult6b1 |
| scaffold_102 | 1,800,001 | 330 | 0.544887 | Ttll1 |
|  |  |  |  | BAK1 |
|  |  |  |  | MCAT |
|  |  |  |  | Tspo |
|  |  |  |  | TTLL12 |
|  |  |  |  | SCUBE1 |
| scaffold_102 | 2,100,001 | 395 | 0.563974 | No genes predicted in this region |
| scaffold_102 | 2,300,001 | 237 | 0.715245 | Sult4a1 |
|  |  |  |  | Pnpla2 |
|  |  |  |  | SAMM50 |
|  |  |  |  | PARVB |
|  |  |  |  | PARVG |
| scaffold_103 | 2,200,001 | 185 | 0.702576 | DHX38 |
|  |  |  |  | DHODH |
|  |  |  |  | IST1 |
|  |  |  |  | ZNF821 |
| scaffold_103 | 2,300,001 | 141 | 0.729693 | AP1G1 |
|  |  |  |  | PHLPP2 |
|  |  |  |  | TAT |
|  |  |  |  | CHST4 |
|  |  |  |  | Kars |
|  |  |  |  | ADAT1 |
|  |  |  |  | Gabarapl2 |
|  |  |  |  | TMEM231 |
|  |  |  |  | CHST5 |
| scaffold_11 | 100,001 | 233 | 0.610186 | COL8A1 |
|  |  |  |  | ST3GAL6 |
| scaffold_11 | 600,001 | 316 | 0.585958 | Slitrk3 |
|  |  |  |  | ATP1B1 |
| scaffold_11 | 700,001 | 288 | 0.66892 | NME7 |
|  |  |  |  | CCDC181 |
|  |  |  |  | BLZF1 |
|  |  |  |  | SLC19A2 |
|  |  |  |  | F5 |
| scaffold_11 | 1,300,001 | 342 | 0.532722 | Apovitellenin-1 |
|  |  |  |  | Gpr15 |
|  |  |  |  | CLDND1 |
|  |  |  |  | PLA2G10 |
|  |  |  |  | Ig kappa chain V-V region HP 91A3 |
|  |  |  |  | GRAMD1C |
|  |  |  |  | Zdhhc23 |
|  |  |  |  | CCDC191 |
|  |  |  |  | QTRT2 |
|  |  |  |  | Drd3 |
| scaffold_115 | 200,001 | 236 | 0.798058 | UGT8 |
| scaffold_115 | 300,001 | 209 | 0.777937 | ARSJ |
| scaffold_115 | 400,001 | 238 | 0.740728 | No genes predicted in this region |
| scaffold_115 | 500,001 | 226 | 0.742673 | CAMK2D |
|  |  |  |  | NPFFR2 |
|  |  |  |  | OVGP1 |
|  |  |  |  | ADAMTS3 |
| scaffold_115 | 600,001 | 260 | 0.620838 | Cox18 |
| scaffold_115 | 700,001 | 235 | 0.628877 | ANKRD17 |
|  |  |  |  | ALB |
| scaffold_115 | 800,001 | 302 | 0.584371 | RASSF6 |
|  |  |  |  | FRAS1 |
| scaffold_115 | 900,001 | 243 | 0.673404 | MRPL1 |
|  |  |  |  | CNOT6L |
|  |  |  |  | MDV078 |
|  |  |  |  | CXCL5 |
|  |  |  |  | CCNG2 |
| scaffold_115 | 1,000,001 | 279 | 0.5379 | CCNI |
|  |  |  |  | SEPT11 |
|  |  |  |  | SOWAHB |
|  |  |  |  | SHROOM3 |
| scaffold_115 | 1,800,001 | 418 | 0.594823 | PCDH18 |
|  |  |  |  | Mroh7 |
| scaffold_120 | 1 | 587 | 0.532566 | No genes predicted in this region |
| scaffold_120 | 100,001 | 404 | 0.562177 | No genes predicted in this region |
| scaffold_120 | 1,900,001 | 356 | 0.526701 | WWC2 |
|  |  |  |  | CLDN22 |
|  |  |  |  | Cdkn2aip |
|  |  |  |  | Ing2 |
| scaffold_122 | 1,800,001 | 252 | 0.5786 | ARL16 |
|  |  |  |  | Hgs |
|  |  |  |  | Mrpl12 |
|  |  |  |  | Slc25a10 |
|  |  |  |  | Gcgr |
|  |  |  |  | Ppp1r27 |
|  |  |  |  | P4HB |
|  |  |  |  | Arhgdia |
|  |  |  |  | ALYREF |
|  |  |  |  | ANAPC11 |
|  |  |  |  | PCYT2 |
|  |  |  |  | Sirt7 |
|  |  |  |  | MAFG |
|  |  |  |  | Pycr2 |
|  |  |  |  | MYADML2 |
|  |  |  |  | Notum |
|  |  |  |  | ASPSCR1 |
|  |  |  |  | LRRC45 |
|  |  |  |  | Rac3 |
|  |  |  |  | RFNG |
|  |  |  |  | csn1 |
|  |  |  |  | Dus1l |
| scaffold_127 | 100,001 | 221 | 0.565669 | Dvl1 |
|  |  |  |  | TAS1R3 |
|  |  |  |  | CPTP |
|  |  |  |  | CPSF3L |
|  |  |  |  | pusl1 |
|  |  |  |  | ACAP3 |
|  |  |  |  | SCNN1A |
|  |  |  |  | UBE2J2 |
|  |  |  |  | fam132a |
|  |  |  |  | B3galt6 |
|  |  |  |  | SDF4 |
|  |  |  |  | Tnfrsf4 |
|  |  |  |  | Tnfrsf21 |
|  |  |  |  | C1orf159 |
| scaffold_14 | 2,200,001 | 315 | 0.531556 | POU3F2 |
| scaffold_14 | 2,300,001 | 336 | 0.571851 | no genes predicted in this region |
| scaffold_14 | 5,600,001 | 305 | 0.535152 | MAP3K7 |
| scaffold_142 | 1,500,001 | 151 | 0.595382 | FAM53A |
|  |  |  |  | Slbp |
| scaffold_149 | 1,100,001 | 221 | 0.53089 | ARFGEF2 |
|  |  |  |  | PREX1 |
| scaffold_149 | 1,300,001 | 193 | 0.614961 | SULF2 |
|  |  |  |  | NCOA3 |
| scaffold_155 | 700,001 | 495 | 0.605012 | RGCC |
|  |  |  |  | si:dkey-18l1.1 |
| scaffold_156 | 100,001 | 234 | 0.618024 | KDM3B |
|  |  |  |  | ctbp2 |
|  |  |  |  | GFRA4 |
|  |  |  |  | CDC23 |
|  |  |  |  | JAKMIP2 |
|  |  |  |  | Dpysl3 |
|  |  |  |  | Stk32a |
| scaffold_17 | 10,200,001 | 348 | 0.531086 | DPF3 |
|  |  |  |  | RGS6 |
| scaffold_174 | 100,001 | 204 | 0.599254 | VPS11 |
|  |  |  |  | DDX6 |
|  |  |  |  | Cxcr5 |
|  |  |  |  | BCL9L |
|  |  |  |  | CCDC84 |
|  |  |  |  | RPS25 |
|  |  |  |  | TRAPPC4 |
|  |  |  |  | SLC37A4 |
|  |  |  |  | HYOU1 |
|  |  |  |  | PHLDB1 |
|  |  |  |  | ARCN1 |
|  |  |  |  | IFT46 |
|  |  |  |  | TMEM25 |
|  |  |  |  | TTC36 |
|  |  |  |  | KMT2A |
| scaffold_176 | 100,001 | 238 | 0.565302 | MARK1 |
|  |  |  |  | MARC2 |
|  |  |  |  | dsbD |
|  |  |  |  | Hlx |
|  |  |  |  | SCNN1G |
| scaffold_176 | 500,001 | 180 | 0.605658 | DUSP10 |
| scaffold_19 | 2,900,001 | 396 | 0.528041 | BCLAF1 |
|  |  |  |  | vac8 |
|  |  |  |  | MAP7 |
|  |  |  |  | MAP3K5 |
| scaffold_206 | 1 | 504 | 0.553355 | No genes predicted in this region |
| scaffold_206 | 100,001 | 354 | 0.622965 | AGA |
| scaffold_206 | 300,001 | 274 | 0.656224 | NEIL3 |
| scaffold_206 | 400,001 | 253 | 0.777602 | VEGFC |
| scaffold_206 | 500,001 | 207 | 0.799962 | SPC22 |
| scaffold_206 | 600,001 | 277 | 0.661355 | ASB5 |
|  |  |  |  | WDR17 |
| scaffold_206 | 800,001 | 117 | 0.816479 | GPM6A |
| scaffold_215 | 1 | 220 | 0.711096 | NTF3 |
| scaffold_215 | 100,001 | 238 | 0.693515 | ANO2 |
| scaffold_215 | 300,001 | 208 | 0.733007 | VWF |
|  |  |  |  | Cd9 |
|  |  |  |  | Nobox |
| scaffold_221 | 500,001 | 305 | 0.627799 | No genes predicted in this region |
| scaffold_221 | 700,001 | 173 | 0.545519 | Cd96 |
| scaffold_222 | 1 | 306 | 0.599721 |  |
| scaffold_23 | 8,800,001 | 347 | 0.601362 | MDH1 |
|  |  |  |  | UGP2 |
|  |  |  |  | VPS54 |
|  |  |  |  | PELI1 |
| scaffold_242 | 1 | 224 | 0.621413 | No genes predicted in this region |
| scaffold_242 | 200,001 | 196 | 0.615395 | NDUFA9 |
|  |  |  |  | Kcna6 |
|  |  |  |  | KCNA1 |
| scaffold_246 | 500,001 | 319 | 0.533491 | LHX8 |
|  |  |  |  | SLC44A5 |
|  |  |  |  | ACADM |
|  |  |  |  | RABGGTB |
| scaffold_261 | 1 | 368 | 0.534709 | GPN1 |
|  |  |  |  | ZNF512 |
|  |  |  |  | Fndc4 |
|  |  |  |  | Ldlrap1 |
|  |  |  |  | Ift172 |
|  |  |  |  | NRBP1 |
|  |  |  |  | KRTCAP3 |
| scaffold_27 | 5,500,001 | 337 | 0.588664 | IFI6 |
|  |  |  |  | SEC61B |
|  |  |  |  | ALG2 |
| scaffold_28 | 9,900,001 | 302 | 0.564195 | ACR |
|  |  |  |  | Slc30a10 |
|  |  |  |  | GluProRS |
|  |  |  |  | BPNT1 |
|  |  |  |  | IARS2 |
| scaffold_28 | 10,000,001 | 144 | 0.604865 | RAB3GAP2 |
| scaffold_30 | 7,000,001 | 275 | 0.545711 | FGF5 |
|  |  |  |  | PRDM8 |
|  |  |  |  | FAT4 |
|  |  |  |  | ANTXR2 |
| scaffold_30 | 7,100,001 | 311 | 0.539682 | No genes predicted in this region |
| scaffold_30 | 7,500,001 | 369 | 0.551837 | CXCL8 |
|  |  |  |  | MTHFD2L |
|  |  |  |  | EPGN |
|  |  |  |  | EREG |
|  |  |  |  | AREG |
|  |  |  |  | Uso1 |
| scaffold_30 | 8,100,001 | 325 | 0.535057 | Cdkl2 |
|  |  |  |  | RCHY1 |
|  |  |  |  | Parm1 |
|  |  |  |  | BTC |
|  |  |  |  | DCTN6 |
|  |  |  |  | RBPMS |
|  |  |  |  | GTF2E2 |
|  |  |  |  | WRN |
|  |  |  |  | PURG |
|  |  |  |  | Gsr |
| scaffold_30 | 8,300,001 | 310 | 0.56096 | SLC4A4 |
|  |  |  |  | DCK |
| scaffold_30 | 8,400,001 | 309 | 0.528788 | Mob1b |
|  |  |  |  | GRSF1 |
|  |  |  |  | RUFY3 |
|  |  |  |  | Utp3 |
|  |  |  |  | Jchain |
|  |  |  |  | SDAD1 |
|  |  |  |  | NAAA |
|  |  |  |  | PPEF2 |
|  |  |  |  | Nup54 |
|  |  |  |  | SCARB2 |
| scaffold_30 | 8,600,001 | 291 | 0.596326 | folD |
|  |  |  |  | Noct |
|  |  |  |  | ELF2 |
|  |  |  |  | MGARP |
|  |  |  |  | NAA15 |
|  |  |  |  | RAB33B |
|  |  |  |  | fadR |
|  |  |  |  | SETD7 |
| scaffold_30 | 8,900,001 | 258 | 0.559292 | SCOC |
|  |  |  |  | CLGN |
|  |  |  |  | mgat4b |
|  |  |  |  | ELMOD2 |
|  |  |  |  | TBC1D9 |
| scaffold_30 | 9,300,001 | 388 | 0.609743 | INPP4B |
| scaffold_33 | 600,001 | 380 | 0.535356 | SS18 |
| scaffold_33 | 2,100,001 | 440 | 0.528815 | GATA6 |
|  |  |  |  | MIB1 |
| scaffold_35 | 4,500,001 | 830 | 0.530141 | epabp |
|  |  |  |  | Tomm34 |
|  |  |  |  | STK4 |
|  |  |  |  | ypiA |
|  |  |  |  | KCNS1 |
|  |  |  |  | MATN4 |
|  |  |  |  | RBPJL |
|  |  |  |  | SDC4 |
|  |  |  |  | SYS1 |
|  |  |  |  | nrn1 |
|  |  |  |  | DTNBP1 |
|  |  |  |  | PIGT |
| scaffold_37 | 1 | 621 | 0.527499 | ZAN |
|  |  |  |  | MB21D2 |
| scaffold_38 | 7,100,001 | 466 | 0.601724 | Gpr83 |
|  |  |  |  | EDA2R |
|  |  |  |  | AR |
|  |  |  |  | OPHN1 |
|  |  |  |  | yipf6 |
| scaffold_44 | 5,600,001 | 273 | 0.566153 |  |
| scaffold_44 | 5,700,001 | 209 | 0.721474 | CRY1 |
|  |  |  |  | MTERF2 |
|  |  |  |  | TMEM263 |
| scaffold_44 | 5,800,001 | 259 | 0.587861 | Ric8b |
|  |  |  |  | RFX4 |
| scaffold_44 | 6,000,001 | 275 | 0.534607 | POLR3B |
|  |  |  |  | TCP11L2 |
|  |  |  |  | CKAP4 |
|  |  |  |  | NUAK1 |
|  |  |  |  | tra-2 |
| scaffold_44 | 6,400,001 | 229 | 0.766646 | APPL2 |
|  |  |  |  | WASHC4 |
|  |  |  |  | ALDH1L2 |
|  |  |  |  | D10Wsu102e |
|  |  |  |  | SLC41A2 |
|  |  |  |  | CHST11 |
| scaffold_44 | 6,600,001 | 162 | 0.779575 | TXNRD1 |
| scaffold_5 | 16,500,001 | 486 | 0.529322 |  |
| scaffold_6 | 6,500,001 | 373 | 0.529106 | SLCO5A1 |
| scaffold_6 | 12,900,001 | 491 | 0.531781 | RB1CC1 |
| scaffold_6 | 14,800,001 | 578 | 0.535903 | UBE2V2 |
|  |  |  |  | MCM4 |
|  |  |  |  | PRKDC |
| scaffold_62 | 100,001 | 159 | 0.646623 | flgH |
|  |  |  |  | FGD5 |
|  |  |  |  | NR2C2 |
|  |  |  |  | MRPS25 |
|  |  |  |  | RBSN |
|  |  |  |  | TRH |
| scaffold_63 | 100,001 | 470 | 0.539515 | MRE11 |
|  |  |  |  | GPR83 |
|  |  |  |  | PANX1 |
|  |  |  |  | HEPHL1 |
|  |  |  |  | TM4SF1 |
|  |  |  |  | Slc5a7 |
|  |  |  |  | VSTM5 |
|  |  |  |  | MED17 |
|  |  |  |  | Kctd5 |
|  |  |  |  | C11orf54 |
|  |  |  |  | Taf1d |
|  |  |  |  | CEP295 |
| scaffold_8 | 100,001 | 230 | 0.5873 | Pdpk1 |
|  |  |  |  | IL21R |
|  |  |  |  | nsmce1 |
|  |  |  |  | Kdm8 |
|  |  |  |  | Serinc3 |

**Table S4:** Windows exhibiting elevated divergence between saltmarsh and Nelson’s sparrows, assessed as regions with F*_ST_* estimates greater than the 99.5^th^ percentile of the empirical distribution. Table includes information on the region exhibiting elevated F*_ST_* estimates (scaffold and window start position), the number of SNPs contained within each window, mean F*_ST_*, and MAKER annotations for genes contained within 50 kb of the windows. Genes that have a hypothesized role in bill morphology are highlighted in red, those that have a hypothesized role in tidal marsh adaptations are highlighted in green, genes with hypothesized roles in circadian rhythm regulation are highlighted in blue, and genes with other potential adaptive functions are in grey.

| **Scaffold** | **Window Starting Position** | **Number of Variants in Window** | **Mean Fst** | **Maker Annotation** |
| --- | --- | --- | --- | --- |
| scaffold_10 | 1,300,001 | 657 | 0.602402 | ANXA8 |
|  |  |  |  | NPY4R |
|  |  |  |  | MSMB |
|  |  |  |  | WASHC2C |
|  |  |  |  | Zfand4 |
| scaffold_102 | 2,300,001 | 237 | 0.715245 | Sult4a1 |
|  |  |  |  | Pnpla2 |
|  |  |  |  | SAMM50 |
|  |  |  |  | PARVB |
|  |  |  |  | PARVG |
| scaffold_103 | 2,200,001 | 185 | 0.702576 | DHX38 |
|  |  |  |  | DHODH |
|  |  |  |  | IST1 |
|  |  |  |  | ZNF821 |
| scaffold_103 | 2,300,001 | 141 | 0.729693 | AP1G1 |
|  |  |  |  | PHLPP2 |
|  |  |  |  | TAT |
|  |  |  |  | CHST4 |
|  |  |  |  | Kars |
|  |  |  |  | ADAT1 |
|  |  |  |  | Gabarapl2 |
|  |  |  |  | TMEM231 |
|  |  |  |  | CHST5 |
| scaffold_11 | 100,001 | 233 | 0.610186 | COL8A1 |
|  |  |  |  | ST3GAL6 |
| scaffold_11 | 600,001 | 316 | 0.585958 | Slitrk3 |
|  |  |  |  | ATP1B1 |
| scaffold_11 | 700,001 | 288 | 0.66892 | NME7 |
|  |  |  |  | CCDC181 |
|  |  |  |  | BLZF1 |
|  |  |  |  | SLC19A2 |
|  |  |  |  | F5 |
| scaffold_115 | 200,001 | 236 | 0.798058 | UGT8 |
| scaffold_115 | 300,001 | 209 | 0.777937 | ARSJ |
| scaffold_115 | 400,001 | 238 | 0.740728 | No genes predicted in this region |
| scaffold_115 | 500,001 | 226 | 0.742673 | CAMK2D |
|  |  |  |  | NPFFR2 |
|  |  |  |  | OVGP1 |
|  |  |  |  | ADAMTS3 |
| scaffold_115 | 600,001 | 260 | 0.620838 | Cox18 |
| scaffold_115 | 700,001 | 235 | 0.628877 | ANKRD17 |
|  |  |  |  | ALB |
| scaffold_115 | 900,001 | 243 | 0.673404 | MRPL1 |
|  |  |  |  | CNOT6L |
|  |  |  |  | MDV078 |
|  |  |  |  | CXCL5 |
|  |  |  |  | CCNG2 |
| scaffold_115 | 1,800,001 | 418 | 0.594823 | PCDH18 |
|  |  |  |  | Mroh7 |
| scaffold_142 | 1,500,001 | 151 | 0.595382 | FAM53A |
|  |  |  |  | Slbp |
| scaffold_149 | 1,300,001 | 193 | 0.614961 | SULF2 |
|  |  |  |  | NCOA3 |
| scaffold_155 | 700,001 | 495 | 0.605012 | RGCC |
|  |  |  |  | si:dkey-18l1.1 |
| scaffold_156 | 100,001 | 234 | 0.618024 | KDM3B |
|  |  |  |  | ctbp2 |
|  |  |  |  | GFRA4 |
|  |  |  |  | CDC23 |
|  |  |  |  | JAKMIP2 |
|  |  |  |  | Dpysl3 |
|  |  |  |  | Stk32a |
| scaffold_174 | 100,001 | 204 | 0.599254 | VPS11 |
|  |  |  |  | DDX6 |
|  |  |  |  | Cxcr5 |
|  |  |  |  | BCL9L |
|  |  |  |  | CCDC84 |
|  |  |  |  | RPS25 |
|  |  |  |  | TRAPPC4 |
|  |  |  |  | SLC37A4 |
|  |  |  |  | HYOU1 |
|  |  |  |  | PHLDB1 |
|  |  |  |  | ARCN1 |
|  |  |  |  | IFT46 |
|  |  |  |  | TMEM25 |
|  |  |  |  | TTC36 |
|  |  |  |  | KMT2A |
| scaffold_176 | 500,001 | 180 | 0.605658 | DUSP10 |
| scaffold_206 | 100,001 | 354 | 0.622965 | AGA |
| scaffold_206 | 300,001 | 274 | 0.656224 | NEIL3 |
| scaffold_206 | 400,001 | 253 | 0.777602 | VEGFC |
| scaffold_206 | 500,001 | 207 | 0.799962 | SPC22 |
| scaffold_206 | 600,001 | 277 | 0.661355 | ASB5 |
|  |  |  |  | WDR17 |
| scaffold_206 | 800,001 | 117 | 0.816479 | GPM6A |
| scaffold_215 | 1 | 220 | 0.711096 | NTF3 |
| scaffold_215 | 100,001 | 238 | 0.693515 | ANO2 |
| scaffold_215 | 300,001 | 208 | 0.733007 | VWF |
|  |  |  |  | Cd9 |
|  |  |  |  | Nobox |
| scaffold_221 | 500,001 | 305 | 0.627799 | No genes predicted in this region |
| scaffold_222 | 1 | 306 | 0.599721 | No genes predicted in this region |
| scaffold_23 | 8,800,001 | 347 | 0.601362 | MDH1 |
|  |  |  |  | UGP2 |
|  |  |  |  | VPS54 |
|  |  |  |  | PELI1 |
| scaffold_242 | 1 | 224 | 0.621413 | No genes predicted in this region |
| scaffold_242 | 200,001 | 196 | 0.615395 | NDUFA9 |
|  |  |  |  | Kcna6 |
|  |  |  |  | KCNA1 |
| scaffold_27 | 5,500,001 | 337 | 0.588664 | IFI6 |
|  |  |  |  | SEC61B |
|  |  |  |  | ALG2 |
| scaffold_28 | 10,000,001 | 144 | 0.604865 | RAB3GAP2 |
|  |  |  |  | GluProRS |
|  |  |  |  | BPNT1 |
|  |  |  |  | IARS2 |
| scaffold_30 | 8,600,001 | 291 | 0.596326 | folD |
|  |  |  |  | Noct |
|  |  |  |  | ELF2 |
|  |  |  |  | MGARP |
|  |  |  |  | NAA15 |
|  |  |  |  | RAB33B |
|  |  |  |  | fadR |
|  |  |  |  | SETD7 |
| scaffold_30 | 9,300,001 | 388 | 0.609743 | INPP4B |
| scaffold_38 | 7,100,001 | 466 | 0.601724 | Gpr83 |
|  |  |  |  | EDA2R |
|  |  |  |  | AR |
|  |  |  |  | OPHN1 |
|  |  |  |  | yipf6 |
| scaffold_44 | 5,700,001 | 209 | 0.721474 | CRY1 |
|  |  |  |  | MTERF2 |
|  |  |  |  | TMEM263 |
| scaffold_44 | 5,800,001 | 259 | 0.587861 | Ric8b |
|  |  |  |  | RFX4 |
| scaffold_44 | 6,400,001 | 229 | 0.766646 | APPL2 |
|  |  |  |  | WASHC4 |
|  |  |  |  | ALDH1L2 |
|  |  |  |  | D10Wsu102e |
|  |  |  |  | SLC41A2 |
|  |  |  |  | CHST11 |
| scaffold_44 | 6,600,001 | 162 | 0.779575 | TXNRD1 |
| scaffold_62 | 100,001 | 159 | 0.646623 | flgH |
|  |  |  |  | FGD5 |
|  |  |  |  | NR2C2 |
|  |  |  |  | MRPS25 |
|  |  |  |  | RBSN |
|  |  |  |  | TRH |
| scaffold_8 | 100,001 | 230 | 0.5873 | Pdpk1 |
|  |  |  |  | IL21R |
|  |  |  |  | nsmce1 |
|  |  |  |  | Kdm8 |
|  |  |  |  | Serinc3 |

**Table S5:** Windows exhibiting elevated divergence between saltmarsh and Nelson’s sparrows, assessed as regions containing numbers of fixed SNPs that are higher than the 99^th^ percentile of the empirical distribution. Table includes information on the region exhibiting elevated F*_ST_* estimates (scaffold and window start position), the number of SNPs contained within each window, mean F*_ST_*, and MAKER annotations for genes contained within 50 kb of the windows. Genes that have a hypothesized role in bill morphology or plumage are highlighted in red and orange, respectively, those that have a hypothesized role in tidal marsh adaptations are highlighted in green, genes with hypothesized roles in circadian rhythm regulation are highlighted in blue, and genes with other potential adaptive functions are in grey.

| **Scaffold** | **Window Starting Position** | **Number of Fixed SNPs** | **Maker Annotation** |
| --- | --- | --- | --- |
| 0 | 9,800,001 | 150 | No genes predicted in this region |
| 0 | 10,000,001 | 150 | No genes predicted in this region |
| 0 | 32,900,001 | 190 | LRRFIP1 |
|  |  |  | Rab17 |
|  |  |  | MLPH |
|  |  |  | COL6A3 |
| 0 | 33,000,001 | 153 | Mroh7 |
| 4 | 19,500,001 | 131 | No genes predicted in this region |
| 5 | 12,700,001 | 141 | No genes predicted in this region |
| 5 | 18,500,001 | 139 | No genes predicted in this region |
| 5 | 18,700,001 | 158 | No genes predicted in this region |
| 5 | 18,900,001 | 135 | No genes predicted in this region |
| 5 | 19,400,001 | 129 | Ccdc82 |
| 6 | 5,900,001 | 143 | EYA1 |
|  |  |  | lactb2 |
| 6 | 12,800,001 | 175 | Oprk1 |
|  |  |  | RB1CC1 |
| 6 | 13,100,001 | 158 | ST18 |
| 6 | 13,900,001 | 147 | No genes predicted in this region |
| 6 | 14,800,001 | 151 | UBE2V2 |
|  |  |  | mcm4 |
|  |  |  | PRKDC |
| 8 | 12,700,001 | 143 | No genes predicted in this region |
| 10 | 1,300,001 | 223 | ANXA8 |
|  |  |  | NPY4R |
|  |  |  | MSMB |
|  |  |  | WASHC2C |
|  |  |  | Zfand4 |
| 11 | 200,001 | 138 | COL8A1 |
|  |  |  | ST3GAL6 |
|  |  |  | CPOX |
|  |  |  | TTF2 |
|  |  |  | TIPRL |
|  |  |  | SFT2D2 |
| 11 | 300,001 | 130 | TBXT |
| 11 | 600,001 | 144 | Slitrk3 |
|  |  |  | ATP1B1 |
|  |  |  | NME7 |
| 11 | 700,001 | 144 | CCDC181 |
|  |  |  | BLZF1 |
|  |  |  | SLC19A2 |
|  |  |  | F5 |
| 11 | 6,200,001 | 140 | CD86 |
|  |  |  | Casr |
|  |  |  | CSTB |
|  |  |  | CCDC58 |
|  |  |  | KPNA1 |
| 12 | 200,001 | 135 | KIAA1462 |
| 12 | 3,100,001 | 137 | No genes predicted in this region |
| 14 | 1 | 133 | No genes predicted in this region |
| 14 | 1,500,001 | 130 | No genes predicted in this region |
| 14 | 3,100,001 | 134 | Kif11 |
|  |  |  | NDUFAF4 |
|  |  |  | GPR63 |
|  |  |  | Fhl5 |
| 14 | 3,300,001 | 128 | UFL1 |
|  |  |  | Fut9 |
| 19 | 600,001 | 132 | SASH1 |
| 19 | 5,000,001 | 139 | NMBR |
|  |  |  | Gje1 |
|  |  |  | VTA1 |
| 21 | 9,300,001 | 131 | SLC32A1 |
| 28 | 4,500,001 | 128 | MTA3 |
|  |  |  | Kcng3 |
|  |  |  | COX7A2L |
| 29 | 9,500,001 | 146 | No genes predicted in this region |
| 30 | 9,300,001 | 162 | INPP4B |
| 33 | 4,800,001 | 127 | TMEM200C |
| 33 | 8,800,001 | 154 | No genes predicted in this region |
| 33 | 8,900,001 | 139 | No genes predicted in this region |
| 35 | 4,500,001 | 203 | epabp |
|  |  |  | Tomm34 |
|  |  |  | STK4 |
|  |  |  | ypiA |
|  |  |  | KCNS1 |
|  |  |  | MATN4 |
|  |  |  | RBPJL |
|  |  |  | SDC4 |
|  |  |  | SYS1 |
|  |  |  | nrn1 |
|  |  |  | DTNBP1 |
|  |  |  | PIGT |
| 38 | 7,100,001 | 184 | Gpr83 |
|  |  |  | EDA2R |
|  |  |  | AR |
|  |  |  | OPHN1 |
|  |  |  | yipf6 |
| 44 | 5,700,001 | 131 | CRY1 |
|  |  |  | MTERF2 |
|  |  |  | TMEM263 |
|  |  |  | Ric8b |
|  |  |  | RFX4 |
| 44 | 6,400,001 | 156 | APPL2 |
|  |  |  | WASHC4 |
|  |  |  | ALDH1L2 |
|  |  |  | D10Wsu102e |
|  |  |  | SLC41A2 |
|  |  |  | CHST11 |
| 47 | 5,800,001 | 137 | No genes predicted in this region |
| 54 | 1 | 293 | SERPINB10 |
|  |  |  | SERPINB14 |
|  |  |  | SERPINB14B |
|  |  |  | SERPINB5 |
| 70 | 2,500,001 | 150 | PCDH11X |
| 71 | 800,001 | 129 | No genes predicted in this region |
| 80 | 800,001 | 159 | CMTM3 |
|  |  |  | Cmtm4 |
|  |  |  | Dync1li2 |
|  |  |  | TERB1 |
|  |  |  | HYDIN |
|  |  |  | NAE1 |
|  |  |  | CA7 |
| 91 | 300,001 | 127 | WNT4 |
| 91 | 400,001 | 149 | No genes predicted in this region |
| 93 | 800,001 | 133 | EXOC6B |
| 102 | 2,000,001 | 146 | SCUBE1 |
| 102 | 2,100,001 | 132 | No genes predicted in this region |
| 115 | 200,001 | 165 | UGT8 |
| 115 | 300,001 | 148 | ARSJ |
| 115 | 400,001 | 155 | SPs1787 |
| 115 | 500,001 | 147 | CAMK2D |
|  |  |  | SPs1787 |
|  |  |  | CAMK2D |
|  |  |  | NPFFR2 |
|  |  |  | ADAMTS3 |
| 115 | 700,001 | 127 | Cox18 |
|  |  |  | ANKRD17 |
|  |  |  | ALB |
| 115 | 800,001 | 129 | RASSF6 |
|  |  |  | FRAS1 |
| 115 | 900,001 | 130 | MRPL1 |
|  |  |  | CNOT6L |
|  |  |  | MDV078 |
|  |  |  | Cxcl5 |
|  |  |  | CCNG2 |
|  |  |  | CCNI |
|  |  |  | SEPT11 |
| 115 | 1,300,001 | 146 | No genes predicted in this region |
| 115 | 1,400,001 | 155 | SLC7A11 |
| 115 | 1,700,001 | 144 | PCDH18 |
| 115 | 1,800,001 | 173 | PCDH18 |
|  |  |  | Mroh7 |
| 120 | 1 | 154 | No genes predicted in this region |
| 120 | 1,100,001 | 144 | No genes predicted in this region |
| 149 | 900,001 | 164 | B4GALT5 |
|  |  |  | KCNB1 |
|  |  |  | STAU1 |
|  |  |  | CSE1L |
| 155 | 700,001 | 176 | si:dkey-18l1.1 |
|  |  |  | RGCC |
| 158 | 1,100,001 | 154 | No genes predicted in this region |
| 170 | 100,001 | 133 | No genes predicted in this region |
| 177 | 200,001 | 127 | No genes predicted in this region |
|  |  |  | No genes predicted in this region |
| 181 | 300,001 | 129 | IRF2 |
|  |  |  | CASP3 |
|  |  |  | primpol |
|  |  |  | CENPU |
|  |  |  | Acsl1 |
|  |  |  | HELT |
| 183 | 1 | 142 | No genes predicted in this region |
| 183 | 200,001 | 172 | No genes predicted in this region |
| 183 | 300,001 | 135 | No genes predicted in this region |
| 183 | 400,001 | 153 | No genes predicted in this region |
| 190 | 400,001 | 134 | Adam11 |
|  |  |  | GJC1 |
|  |  |  | EFTUD2 |
|  |  |  | Ccdc103 |
|  |  |  | FAM187A |
|  |  |  | KIF18B |
|  |  |  | C1ql3 |
|  |  |  | dcakd |
|  |  |  | NMT1 |
|  |  |  | PLCD3 |
| 190 | 600,001 | 207 | Mylk |
|  |  |  | FMNL1 |
|  |  |  | MAP3K14 |
|  |  |  | MYO1D |
| 190 | 700,001 | 160 | Psmd11 |
| 193 | 600,001 | 135 | C3orf38 |
|  |  |  | ZNF654 |
| 193 | 800,001 | 181 | N6AMT1 |
| 206 | 1 | 163 | No genes predicted in this region |
| 206 | 100,001 | 131 | AGA |
| 206 | 300,001 | 139 | NEIL3 |
| 206 | 400,001 | 153 | VEGFC |
| 206 | 500,001 | 141 | SPC22 |
|  |  |  | Spcs3 |
| 206 | 600,001 | 155 | ASB5 |
|  |  |  | WDR17 |
| 215 | 1 | 131 | NTF3 |
| 215 | 100,001 | 142 | ANO2 |
| 215 | 300,001 | 128 | VWF |
| 215 | 400,001 | 134 | Cd9 |
|  |  |  | Nobox |
|  |  |  | ARHGEF5 |
| 215 | 500,001 | 148 | FAR1 |
| 221 | 500,001 | 153 | No genes predicted in this region |
| 261 | 1 | 141 | GPN1 |
|  |  |  | ZNF512 |
|  |  |  | Fndc4 |
|  |  |  | Ldlrap1 |
|  |  |  | Ift172 |
|  |  |  | KRTCAP3 |
|  |  |  | NRBP1 |
| 294 | 1 | 168 | No genes predicted in this region |

**Table S6:** Windows exhibiting elevated divergence between saltmarsh and Nelson’s sparrows, assessed as regions containing numbers of fixed SNPs that are higher than the 99.5^th^ percentile of the empirical distribution. Table includes information on the region exhibiting elevated F*_ST_* estimates (scaffold and window start position), the number of SNPs contained within each window, mean F*_ST_*, and MAKER annotations for genes contained within 50 kb of the windows. Genes that have a hypothesized role in bill morphology or plumage are highlighted in red and orange, respectively, those that have a hypothesized role in tidal marsh adaptations are highlighted in green, genes with hypothesized roles in circadian rhythm regulation are highlighted in blue, and genes with other potential adaptive functions are in grey.

| **Scaffold** | **Window Starting Position** | **Number of Fixed SNPs** | **Maker Annotation** |
| --- | --- | --- | --- |
| 0 | 9,800,001 | 150 | No genes predicted in this region |
| 0 | 10,000,001 | 150 | No genes predicted in this region |
| 0 | 32,900,001 | 190 | LRRFIP1 |
|  |  |  | Rab17 |
|  |  |  | MLPH |
|  |  |  | COL6A3 |
| 0 | 33,000,001 | 153 | Mroh7 |
| 5 | 18,700,001 | 158 | No genes predicted in this region |
| 6 | 5,900,001 | 143 | EYA1 |
|  |  |  | lactb2 |
| 6 | 12,800,001 | 175 | Oprk1 |
|  |  |  | RB1CC1 |
| 6 | 13,100,001 | 158 | ST18 |
| 6 | 13,900,001 | 147 | No genes predicted in this region |
| 6 | 14,800,001 | 151 | UBE2V2 |
|  |  |  | mcm4 |
|  |  |  | PRKDC |
| 8 | 12,700,001 | 143 | No genes predicted in this region |
| 10 | 1,300,001 | 223 | ANXA8 |
|  |  |  | NPY4R |
|  |  |  | MSMB |
|  |  |  | WASHC2C |
|  |  |  | Zfand4 |
| 11 | 600,001 | 144 | Slitrk3 |
|  |  |  | ATP1B1 |
|  |  |  | NME7 |
| 11 | 700,001 | 144 | CCDC181 |
|  |  |  | BLZF1 |
|  |  |  | SLC19A2 |
|  |  |  | F5 |
| 29 | 9,500,001 | 146 | No genes predicted in this region |
| 30 | 9,300,001 | 162 | INPP4B |
| 33 | 8,800,001 | 154 | No genes predicted in this region |
| 35 | 4,500,001 | 203 | epabp |
|  |  |  | Tomm34 |
|  |  |  | STK4 |
|  |  |  | ypiA |
|  |  |  | KCNS1 |
|  |  |  | MATN4 |
|  |  |  | RBPJL |
|  |  |  | SDC4 |
|  |  |  | SYS1 |
|  |  |  | nrn1 |
|  |  |  | DTNBP1 |
|  |  |  | PIGT |
| 38 | 7,100,001 | 184 | Gpr83 |
|  |  |  | EDA2R |
|  |  |  | AR |
|  |  |  | OPHN1 |
|  |  |  | yipf6 |
| 44 | 6,400,001 | 156 | APPL2 |
|  |  |  | WASHC4 |
|  |  |  | ALDH1L2 |
|  |  |  | D10Wsu102e |
|  |  |  | SLC41A2 |
|  |  |  | CHST11 |
| 54 | 1 | 293 | SERPINB10 |
|  |  |  | SERPINB14 |
|  |  |  | SERPINB14B |
|  |  |  | SERPINB5 |
| 70 | 2,500,001 | 150 | PCDH11X |
| 80 | 800,001 | 159 | Cmtm4 |
|  |  |  | Dync1li2 |
|  |  |  | TERB1 |
|  |  |  | HYDIN |
|  |  |  | NAE1 |
|  |  |  | CA7 |
| 91 | 400,001 | 149 | No genes predicted in this region |
| 102 | 2,000,001 | 146 | SCUBE1 |
| 115 | 200,001 | 165 | UGT8 |
| 115 | 300,001 | 148 | ARSJ |
| 115 | 400,001 | 155 | SPs1787 |
| 115 | 500,001 | 147 | CAMK2D |
|  |  |  | SPs1787 |
|  |  |  | CAMK2D |
|  |  |  | NPFFR2 |
|  |  |  | ADAMTS3 |
| 115 | 1,300,001 | 146 | No genes predicted in this region |
| 115 | 1,400,001 | 155 | SLC7A11 |
| 115 | 1,700,001 | 144 | PCDH18 |
| 115 | 1,800,001 | 173 | PCDH18 |
|  |  |  | Mroh7 |
| 120 | 1 | 154 | No genes predicted in this region |
| 120 | 1,100,001 | 144 | No genes predicted in this region |
| 149 | 900,001 | 164 | B4GALT5 |
|  |  |  | KCNB1 |
|  |  |  | STAU1 |
|  |  |  | CSE1L |
| 155 | 700,001 | 176 | si:dkey-18l1.1 |
|  |  |  | RGCC |
| 158 | 1,100,001 | 154 | No genes predicted in this region |
| 183 | 1 | 142 | No genes predicted in this region |
| 183 | 200,001 | 172 | No genes predicted in this region |
| 183 | 400,001 | 153 | No genes predicted in this region |
| 190 | 600,001 | 207 | Mylk |
|  |  |  | FMNL1 |
|  |  |  | MAP3K14 |
|  |  |  | MYO1D |
| 190 | 700,001 | 160 | Psmd11 |
| 193 | 800,001 | 181 | N6AMT1 |
| 206 | 1 | 163 | No genes predicted in this region |
| 206 | 400,001 | 153 | VEGFC |
| 206 | 600,001 | 155 | ASB5 |
|  |  |  | WDR17 |
| 215 | 500,001 | 148 | FAR1 |
| 221 | 500,001 | 153 | No genes predicted in this region |
| 294 | 1 | 168 | No genes predicted in this region |

**Table S7:** Windows exhibiting elevated divergence, assessed as regions with both F*_ST_* estimates and Df estimates higher than the 99^th^ percentile of the empirical distribution. Table includes information on the regions exhibiting elevated divergence (scaffold and window start position), the number of SNPs contained within each window, mean F*_ST,_* the number of fixed SNPs, MAKER annotations, and biological function. Genes that have putative links to tidal marsh adaptations are in green and genes with other potential adaptive functions are in grey.

| **Scaffold** | **Window Start Position** | **Number of Variants** | **Mean Fst** | **Number of Fixed SNPs** | **Maker Annotation** | **Biological Function** |
| --- | --- | --- | --- | --- | --- | --- |
| scaffold_0 | 9,800,001 | 522 | 0.526282 | 150 | No genes predicted in this region |  |
| scaffold_0 | 10,000,001 | 506 | 0.553127 | 150 | No genes predicted in this region |  |
| scaffold_10 | 1,300,001 | 657 | 0.602402 | 223 | ANXA8 | This protein is an anticoagulant protein that acts as an indirect inhibitor of the thromboplastin-specific complex, which is involved in the blood coagulation cascade |
|  |  |  |  |  | MSMB | Specific receptors for this protein are found on spermatozoa and in the prostate |
|  |  |  |  |  | NPY4R | Blood circulation, chemical synaptic transmission, digestion, feeding behavior, neuropeptide signaling pathway |
|  |  |  |  |  | WASHC2C | negative regulation of barbed-end actin filament capping, protein transport, regulation of substrate adhesion-dependent cell spreading, retrograde transport |
|  |  |  |  |  | Zfand4 | Zinc ion binding. |
| scaffold_102 | 2,100,001 | 395 | 0.563974 | 132 | No genes predicted in this region |  |
| scaffold_11 | 600,001 | 316 | 0.585958 | 144 | ATP1B1 | This is the non-catalytic component of the active enzyme, which catalyzes the hydrolysis of ATP coupled with the exchange of Na+ and K+ ions across the plasma membrane. The beta subunit regulates, through assembly of alpha/beta heterodimers, the number of sodium pumps transported to the plasma membrane |
|  |  |  |  |  | Slitrk3 | Positive regulation of synapse assembly |
| scaffold_11 | 700,001 | 288 | 0.66892 | 144 | BLZF1 | Required for normal Golgi structure and for protein transport from the endoplasmic reticulum (ER) through the Golgi apparatus to the cell surface. |
|  |  |  |  |  | CCDC181 | Coiled-coil domain-containing protein |
|  |  |  |  |  | NME7 | Major role in the synthesis of nucleoside triphosphates other than ATP |
|  |  |  |  |  | SLC19A2 | High-affinity transporter for the intake of thiamine |
|  |  |  |  |  | F5 | Central regulator of hemostasis |
| scaffold_115 | 200,001 | 236 | 0.798058 | 165 | UGT8 | Central nervous system development, cytoskeleton organization, neuron projection morphogenesis, galactosylceramide biosynthetic process, paranodal junction assembly, peripheral nervous system development |
| scaffold_115 | 300,001 | 209 | 0.777937 | 148 | ARSJ | Post translational protein modification, glycosphingolipid metabolic process |
| scaffold_115 | 400,001 | 238 | 0.740728 | 155 | No genes predicted in this region |  |
| scaffold_115 | 500,001 | 226 | 0.742673 | 147 | ADAMTS3 | Collagen biosynthetic process, collagen catabolic process |
|  |  |  |  |  | CAMK2D | Cellular response to calcium ion, MAPK cascade, negative regulation of sodium ion transmembrane transport, nervous system development, regulation of cellular response to heat, calmodulin-dependent protein kinase activity, sodium channel inhibitor activity |
|  |  |  |  |  | NPFFR2 | Cellular response to hormone stimulus, regulation of cAMP-dependent protein kinase activity, regulation of MAPK cascade |
|  |  |  |  |  | OVGP1 | Negative regulation of binding sperm to zona pellucida, single fertilization |
| scaffold_115 | 700,001 | 235 | 0.628877 | 127 | ALB | Sodium-independent organic ion transport, retina homeostasis, bile acid and bile salt transport |
|  |  |  |  |  | ANKRD17 | Blood vessel maturation, defense response to bacterium, innate immune response, negative regulation of smooth muscle cell differentiation, regulation of DNA replication |
| scaffold_115 | 800,001 | 302 | 0.584371 | 129 | FRAS1 | Cell communication, embryonic limb morphogenesis, protein transport, skin development |
|  |  |  |  |  | RASSF6 | apoptotic process |
| scaffold_115 | 900,001 | 243 | 0.673404 | 130 | MRPL1 | Mitochondrial translational elongation and termination |
|  |  |  |  |  | CNOT6L | DNA damage response, gene silencing by RNA, mRNA destabilization, regulation of transcription |
|  |  |  |  |  | MDV078 | Viral gene |
|  |  |  |  |  | CXCL5 | Immune response, inflammatory response |
|  |  |  |  |  | CCNG2 | May play a role in growth regulation and in negative regulation of cell cycle progression |
|  |  |  |  |  | CCNI | Spermatogenesis and regulation of cell cycle |
|  |  |  |  |  | SEPT11 | Cell cycle, cell division |
| scaffold_115 | 1,800,001 | 418 | 0.594823 | 144 | PCDH18 | Brain development, cell adhesion, homophilic cell adhesion, nervous system development |
|  |  |  |  |  | Mroh7 | Unknown |
| scaffold_120 | 1 | 587 | 0.532566 | 154 | None |  |
| scaffold_155 | 700,001 | 495 | 0.605012 | 176 | RGCC | Modulates the activity of cell cycle-specific kinases |
|  |  |  |  |  | si:dkey-18l1.1 | von Willebrand factor A domain-containing protein 8 |
| scaffold_206 | 1 | 504 | 0.553355 | 163 | No genes predicted in this region |  |
| scaffold_206 | 100,001 | 354 | 0.622965 | 131 | AGA | Involved in protein maturation. |
| scaffold_206 | 300,001 | 274 | 0.656224 | 139 | NEIL3 | Nucleotide excision repair |
| scaffold_206 | 400,001 | 253 | 0.777602 | 153 | VEGFC | Growth factor active in angiogenesis, and endothelial cell growth, stimulating their proliferation and migration and also has effects on the permeability of blood vessels. |
| scaffold_206 | 500,001 | 207 | 0.799962 | 141 | SPC22 | Proteolysis, signal peptide processing |
| scaffold_206 | 600,001 | 277 | 0.661355 | 155 | ASB5 | Intracellular signal transduction |
|  |  |  |  |  | WDR17 | WD repeat-containing protein |
| scaffold_215 | 1 | 220 | 0.711096 | 131 | NTF3 | Seems to promote the survival of visceral and proprioceptive sensory neurons. |
| scaffold_215 | 100,001 | 238 | 0.693515 | 142 | ANO2 | Calcium-activated chloride channel (CaCC) which may play a role in olfactory signal transduction |
| scaffold_215 | 300,001 | 208 | 0.733007 | 128 | VWF | Blood coagulation, cell adhesion, hemostasis, platelet activation |
| scaffold_221 | 500,001 | 305 | 0.627799 | 153 | No genes predicted in this region |  |
| scaffold_261 | 1 | 368 | 0.534709 | 141 | Fndc4 | Fibronectin type III domain-containing protein 4 |
|  |  |  |  |  | GPN1 | Small GTPase required for proper nuclear import of RNA polymerase II |
|  |  |  |  |  | Ift172 | Required for the maintenance and formation of cilia |
|  |  |  |  |  | Ldlrap1 | Cholesterol metabolic process |
|  |  |  |  |  | NRBP1 | May play a role in subcellular trafficking between the endoplasmic reticulum and Golgi apparatus through interactions with the Rho-type GTPases. |
|  |  |  |  |  | ZNF512 | May be involved in transcriptional regulation. |
|  |  |  |  |  | KRTCAP3 | Keratinocyte-associated protein 3, integral component of membrane |
| scaffold_30 | 9,300,001 | 388 | 0.609743 | 162 | INPP4B | Signal transduction |
| scaffold_35 | 4,500,001 | 830 | 0.530141 | 203 | epabp | Involved in chordate embryonic development. |
|  |  |  |  |  | Tomm34 | Protein targeting to mitochondrion |
|  |  |  |  |  | STK4 | Key component of the Hippo signaling pathway which plays a pivotal role in organ size control and tumor suppression by restricting proliferation and promoting apoptosis |
|  |  |  |  |  | ypiA | Bacterial gene. |
|  |  |  |  |  | KCNS1 | Potassium ion transport |
|  |  |  |  |  | MATN4 | Major component of the extracellular matrix of cartilage |
|  |  |  |  |  | RBPJL | Positive regulation of transcription |
|  |  |  |  |  | SDC4 | Cell surface proteoglycan that bears heparin sulfate. Regulates exosome biogenesis |
|  |  |  |  |  | SYS1 | Yeast gene. |
|  |  |  |  |  | nrn1 | Promotes neurite outgrowth and especially branching of neuritic processes in primary hippocampal and cortical cells. |
|  |  |  |  |  | DTNBP1 | Regulates dopamine receptor signaling pathway, blood coagulation, melanosome organization and neuronal development. |
|  |  |  |  |  | PIGT | Involved in neuronal differentiation and apoptosis. |
| scaffold_38 | 7,100,001 | 466 | 0.601724 | 184 | Gpr83 | Response to glucocorticoid |
|  |  |  |  |  | EDA2R | Ectodermal cell differentiation, tissue development, epidermis development |
|  |  |  |  |  | AR | Steroid hormone receptors are ligand-activated transcription factors that regulate eukaryotic gene expression and affect cellular proliferation and differentiation in target tissues |
|  |  |  |  |  | OPHN1 | Critical for the regulation of synaptic vesicle endocytosis at presynaptic terminals |
|  |  |  |  |  | yipf6 | integral component of membrane |
|  |  |  |  |  | CRY1 | Transcriptional repressor which forms a core component of the circadian clock |
|  |  |  |  |  | MTERF2 | Binds promoter DNA and regulates mitochondrial transcription. |
|  |  |  |  |  | TMEM263 | Transmembrane protein |
|  |  |  |  |  | Ric8b | G-protein signaling pathway |
|  |  |  |  |  | RFX4 | Plays a role in early brain development. |
| scaffold_44 | 6,400,001 | 229 | 0.766646 | 156 | APPL2 | Platelet degranulation |
|  |  |  |  |  | WASHC4 | Endosomal transport, protein transport |
|  |  |  |  |  | ALDH1L2 | Folic and metabolic process |
|  |  |  |  |  | D10Wsu102e | Uncharacterized. |
|  |  |  |  |  | SLC41A2 | Acts as a plasma-membrane magnesium transporter |
|  |  |  |  |  | CHST11 | Carbohydrate biosynthetic process, developmental growth, embryonic digit morphogenesis, negative regulation of apoptotic process |
| scaffold_6 | 14,800,001 | 578 | 0.535903 | 151 | UBE2V2 | Plays a role in the error-free DNA repair pathway and contributes to the survival of cells after DNA damage |
|  |  |  |  |  | MCM4 | DNA replication initiation, nuclear DNA repair |
|  |  |  |  |  | PRKDC | Serine/threonine-protein kinase that acts as a molecular sensor for DNA damage. |
